# Hierarchical intrinsically motivated agent planning behavior with dreaming in grid environments

**DOI:** 10.1186/s40708-022-00156-6

**Published:** 2022-04-02

**Authors:** Evgenii Dzhivelikian, Artem Latyshev, Petr Kuderov, Aleksandr I. Panov

**Affiliations:** 1 Moscow Institute of Physics and Technology, Dolgoprudny, Russia; 2 Federal Research Center “Computer Science and Control” of the Russian Academy of Sciences, Moscow, Russia; 3 Artificial Intelligence Research Institute (AIRI), Moscow, Russia

**Keywords:** Model-based reinforcement learning, Intrinsic motivation, Hierarchical temporal memory, Sparse distributed representations

## Abstract

Biologically plausible models of learning may provide a crucial insight for building autonomous intelligent agents capable of performing a wide range of tasks. In this work, we propose a hierarchical model of an agent operating in an unfamiliar environment driven by a reinforcement signal. We use temporal memory to learn sparse distributed representation of state–actions and the basal ganglia model to learn effective action policy on different levels of abstraction. The learned model of the environment is utilized to generate an intrinsic motivation signal, which drives the agent in the absence of the extrinsic signal, and through acting in imagination, which we call dreaming. We demonstrate that the proposed architecture enables an agent to effectively reach goals in grid environments.

## Introduction

A defining aspect of intelligence is the ability to accumulate knowledge autonomously and reuse it for a broad range of tasks. Fundamental questions in cognitive sciences include how knowledge is represented in memory, what learning mechanisms exist, and how learning can be self-directed [48]. The studies in animals and humans aim to find a unified biological model of learning and control. Such model could be organized on common mechanisms and principles, which in turn could help us find more effective models of behavior control and, therefore, advance the progress in AI [4, 12].

Reinforcement learning is one of key mechanisms in human learning. In recent years, it has garnered much attention and made progress in the field of AI [43, 56]. While computational reinforcement learning (RL) ultimately aims to solve the same fundamental questions as cognitive sciences do, it does not normally follow biological plausibility, which limits models’ compatibility between these two fields. Moreover, the rapid progress and huge success in practical applications that AI has seen in recent years has only augmented this discrepancy, resulting in deep Artificial Neural Networks (ANNs) with backpropagation-based learning almost monopolizing the field. However, interdisciplinary cooperation may turn out to be vital for more fundamental advances [28]. And RL could serve as the common testbed for computational models of both sides. In our research, we propose a neurophysiologically inspired model of an intelligent agent and apply it to an RL scenario to address the problems of knowledge representation, learning, and motivated behavior.

Humans are proficient at aggregating and reusing their experience in new circumstances and unseen tasks. One aspect that helps us do so is having an inner model of the world around us that we learn and maintain during our lifetime [35, 54]. This internal representation of the external world allows us, among other things, to predict future outcomes of different actions and, therefore, better plan our behavior. We can imagine situations and learn from them. Sometimes, such technique reduces the number of trial-and-error iterations required to successfully complete a new task or improve on it. In our work, we supply an agent with temporal memory, which helps an agent learn a model of the environment. To exploit the learned model and also to test its quality, we additionally supply an agent with the ability to learn in imagination.

There is evidence that behavior and knowledge in humans are organized hierarchically [41, 66]. Such organization allows us to learn and use spatial–temporal abstractions. Computationally, hierarchical organization does not need direct hierarchical structure and can self-arise by subsequent feedforward and recurrent information propagation. In our work, we propose a hierarchical memory model and study how spatial–temporal abstractions help in solving tasks in grid environments.

Another factor that differentiates living creatures from artificial agents is the innate ability to act in the absence of direct goal-related rewarding stimuli [6, 33]. The driving factors that enable such behavior are called intrinsic motivation. Intrinsic motivation plays a fundamental role in developing adaptive and autonomous behavior in animals and humans. We supply our model with the mechanism of generating an intrinsic motivation signal called empowerment. We further supply it with the mechanism that modulates an agent’s behavior by weighting between extrinsic and intrinsic motivations to effectively adapt to changing goals.

Although all aforementioned characteristics are notably inherent to human mind, there is still no generally accepted framework synthesizing them all in an open-ended manner (for example, see a review Parisi et al. [49]). RL systems recently showed significant progress in learning complex behaviors, but there are still many challenges that remain unsolved such as increasing learning and inference time with domain dimensionality, sample efficiency and experience reusability, exploration in domains with high dimensional state–action spaces and sparse rewards, automatic skill acquisition and catastrophic forgetting due to task interference, transfer and lifelong learning [34, 50]. One of the challenges we seek to address by our framework is building a robust general HRL system capable of continuously learning and reusing acquired skills. Therefore, this paper introduces a biologically inspired model of the autonomous agent called HIMA (hierarchical intrinsically motivated agent), which is intended to integrate hierarchical experience organization and intrinsically motivated exploration.

The main feature of our method is combining bottom-up and top-down approaches. That is, on the one hand, we use known neurophysiological computational models of the neocortex and basal ganglia as a starting point and on the other—adapt them for solving RL problem: finding optimal policy given Markov decision process. It is important to follow a biologically plausible course when building an artificial one as stated in Hole and Ahmad [32]. However, we do not consider biological constraints as strictly mandatory. It gives us great flexibility in expanding neurophysiological models according to tasks. Following this way, we have built a decision-making system able to efficiently aggregate and reuse experience for reaching changing goals. We also show that HIMA has much greater flexibility compared to similar DeepRL systems in solving problems that require lifelong continuous learning.

This work builds on preliminary findings presented in the conference paper [22]. This paper provides a more detailed description of our hierarchical memory model than that in the conference paper. We also switched from an anomaly-based to an empowerment-based intrinsic motivation signal. In addition, our model includes a motivational modulation mechanism and the ability to learn in the imagination. Finally, we present a comprehensive experimental analysis of various elements of the proposed model in RL scenarios with changing tasks.

The rest of the paper is organized as follows: Section 2 provides an overview of related works. Section 3 introduces necessary definitions, formalization, and concepts. Section 4 describes our hierarchical memory architecture accompanied with the Basal Ganglia model. We also describe the mechanisms of the generated intrinsic motivation signal, empowerment, and how both an intrinsic and extrinsic motivation signals are modulated to shape behavior. Ultimately, we explain the dreaming ability of an agent. The experimental setup and the results of the experiments performed on a classic grid world environments are described in Sect. 5. Finally, Sects. 6 and 7 discuss the results, outline the proposed method’s limitations, and provide insights for the future work.

## Related works

Learning an inner model of the environment is a distinctive feature of the model-based approach in reinforcement learning [57]. Having a model of the environment allows for an effective combination of planning and learning. The learned model can be utilized to support the learning of an agent’s global policy by supplying it with additional trajectories generated in imagination [58].

Model-based methods can be divided into two groups depending on whether they learn the model and imagine in raw sensory data space [64] or do it in a compact latent space [26, 55]. The former group’s methods usually are simpler to implement and learn, but they require a bigger model. Our method relates to the latter group, partially inspired by the Dreamer [27]. However, in the Dreamer past trajectories are explicitly stored, and the iterative learning process is divided into two separate phases—collecting experience and learning on a sampled data, which includes dreaming. In contrast, our model does not have such separate phases, and an imaginary trajectory during dreaming is allowed to start only from the agent’s current state.

Hierarchical reinforcement learning has extensions that enable operating with non-elementary actions. The Options Framework [5, 60] is among the most popular extensions. It has been hypothesized to be linked with the prefrontal cortex neural structures proposed by Botvinick et al. [9], thereby bridging the gap between the RL model and neurophysiology. Another study discovered that dopamine-driven TD-like learning mechanisms in the dorsal striatum play an important role in the development of a functional hierarchy in the prefrontal cortex [52].

In our work, we draw ideas from a cortical Hierarchical Temporal Memory model [29]. It enables unsupervised hierarchical learning of spatial–temporal data representation. This model, however, has limited utility, as it only defines the elementary building blocks of memory. It does not define either hierarchy, or how to learn temporal abstractions, or how the memory can be integrated into an intelligent agent model. There are works extending its usage in part [21, 30, 38]. However, all of these issues were first to be addressed using an original approach by Dzhivelikian et al. [22]. Our current work is its direct extension, in which we contribute to the analysis of spatial–temporal abstractions arisen in such hierarchy.

Many works are devoted to the problem of autonomy in relation to humans and artificial intelligence agents. One model capable of performing actions even in the absence of an external sensory signal is based on the idea that constant brain activity and self-motivation are innate in living organisms [13]. Another model introduces a causal network and describes the process of maintaining motivation based on a biological representation of the dopamine reward system, which exists in the brain [61]. Santucci and colleagues investigated a variety of intrinsic motivation (IM) models in order to provide autonomy for a robotic agent exploring its surroundings, and the best results were compiled by their GRAIL model [53]. Works by Bolado-Gomez and Gurney [8], Fiore et al. [23] are linked with the similar concept of an agent’s behavior being determined by intrinsic motivation and the interaction of numerous brain components (the cortex, the basal ganglia, the thalamus, the hippocampus, and the amygdala). We were inspired by the GRAIL concept when designing our Basal Ganglia model. However, we constructed the intrinsic motivation mechanism based on the computational model of the empowerment introduced by Klyubin et al. [37]. We also supply our model with soft-gating modulation that enables us to balance between exploratory and exploitatory behavioral programs in order to effectively adapt to the changing goals.

Learning in HER algorithm, proposed by Andrychowicz et al. [2], is an approach that effectively learn how to reach sub-goals based on idea of retrospective learning. However, this method does not extract and learn reusable sub-policies and serves more like curriculum procedure in order to speed up the learning toward the main goal. An extension that combines it with an HRL approach, called Feudal RL [20], addresses this issue by introducing a hierarchy of managers and workers, where workers learn reusable policies [42]. Retrospective learning is not mutually exclusive to our approach and can be seen as a potentially powerful—and biologically plausible [24]—future supplement to the dreaming procedure.

Hierarchical learning inherently imposes the usage of intrinsic motivation for skill acquisition. IM can be used for the better exploration of the sub-goal space as in Antonio Becerra et al. [3], facilitating high-level policy learning. This framework is similar to ours in terms of integrating memory, abstract actions, and intrinsic motivation. However, unlike our work, they use predefined abstract actions and don’t investigate the effects of different IM algorithms. IM can also be used for the task decomposition as in Kulkarni et al. [40]. Although, this method is poorly scalable since it does not have a mechanism for an automatic sub-goal extraction. In Davoodabadi Farahani and Mozayani [19], authors utilize different IM heuristics for both goal discovery and exploration with the Options Framework under-hood. They divide the learning process into two separate stages to overcome intrinsic and extrinsic reward interference, consequently, requiring an explicit indication of a goal change. In contrast, our agent model is capable of identifying a goal change automatically and provides seamless IM and EM integration. Despite the fact that all these frameworks are aiming to integrate IM and HRL, they also differ from HIMA in that they do not explicitly use neurophysiological models that may be an obstacle when interpreting the results in terms of human intelligence.

## Background

This section introduces the definitions and concepts that we will need in our work. We provide formalization in the first subsection that will be used to establish a link between our biologically inspired model and reinforcement learning. Other subsections explain biological concepts and computational models that we use as a foundation.

### MDP, options, and TD

Consider an agent that must make sequential decisions while interacting with an environment. A common approach is to formalize such problem as a Markov Decision Process (MDP) problem: $\langle S, A, P, R, \gamma \rangle$, where *S* is state space, *A* is action space, $P: S\times A \rightarrow S$ is a transition function, $R: S \times A \rightarrow {\mathbb {R}}$ is a reward function, and $\gamma \in [0; 1]$ is a discount factor. Whereas experiment conditions force us to consider the partially observable MDP problem, we can consider $s \in S$ to be an estimate of a function of history of all previous observations. As a result, we use this deterministic MDP formulation throughout the text to simplify derivations.

For actions, we also employ temporal abstractions. The Options Framework is a popular way to generalize both elementary and high-level actions [60]. It defines an option as a tuple $\langle I, \pi , \beta \rangle$, where $I\subseteq S$ is an initiation set, $\pi : S\times A \rightarrow [0, 1]$ is an intra-option policy, and $\beta : S \rightarrow [0, 1]$ is a termination condition. Therefore, a policy over options is a probability function $\mu : S\times O \rightarrow [0, 1]$, where *O* is a set of options.

The agent’s goal is to find such options and policy over options $\mu$ that maximize expected cumulative return:

1 $$\begin{aligned} G_1 = \max _{\mu } {\mathbb {E}}\left[ \,\sum _{t=1}^{\infty }\gamma ^{t-1}r_t\,|\,\mu , O\,\right] . \end{aligned}$$

We use the Temporal Difference Learning [59] to learn the value function. This method has also proven to be biologically plausible [46]. The state value is defined as:

2 $$\begin{aligned} \begin{aligned} v_\pi (s)&= {\mathbb {E}}_\pi \left[ G_t| s_t = s\right] \\&= {\mathbb {E}}_\pi \left[ r_{t+1} + \gamma G_{t+1}| s_t = s\right] \\&= {\mathbb {E}}_\pi \left[ r_{t+1} + \gamma v_\pi (S_{t+1})| s_t = s\right] , \end{aligned} \end{aligned}$$

where $r_t, G_t$ are a reward and a return on *t* timestep. In this approach, the estimate of the value is updated according to the difference between current value $v(s_t)$ and its estimate bootstrapped from the value of the next observed state: ${\hat{v}}(s_t) = r_{t+1} + \gamma v_\pi (s_{t+1})$. This difference is called 1-step TD error: $\delta _t = r_{t+1} + \gamma v(s_{t+1}) - v(s_t)$. Thus, the value update rule is:

3 $$\begin{aligned} v(s_t) \leftarrow v(s_t) + \alpha \delta _t. \end{aligned}$$

### Hierarchical temporal memory

**Fig. 1:**
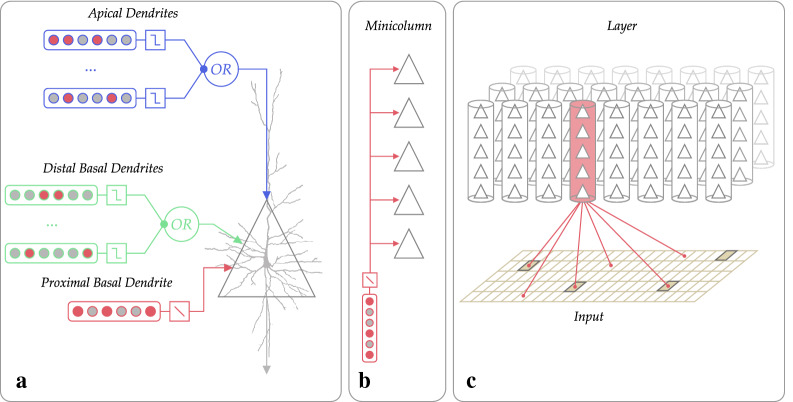
Hierarchical temporal memory framework. **A** HTM neuron. **B** A group of neurons organized into a minicolumn. Neurons within a minicolumn share the same receptive field. **C** A group of minicolumns organized into a layer. Columns within a layer share the same feedforward input, however, they may have different receptive fields

In our memory model, we use the hierarchical temporal memory (HTM) framework proposed by Hawkins and Ahmad [29]. At its core is the model of a discrete-time spiking pyramidal neuron (Fig. 1).

In this model, pyramidal neurons communicate via synapses called active. There are also a special kind of fictive synapses that denote potential connections whose strength is under a specified threshold, so they cannot propagate signals. However, these inactive synapses are subject to learning, so they may become active (and vice versa) in the aftermath. The activation of the presynaptic cell causes a binary spike, which is propagated further through active synapses. Thus, all inputs and outputs of the model are represented as binary patterns.

Dendritic synapses are organized into groups called segments (Fig. 1a). Each neuron has one proximal basal segment and any number of distal basal and apical dendritic segments. A segment defines an activation unit—each segment becomes active independently based on the activity of its receptive field.

Pyramidal neurons are organized into groups called minicolumns (Fig. 1b). Neurons within a minicolumn share the same feedforward (i.e., proximal basal) receptive field, i.e., it is defined by the single proximal basal segment. Therefore, neurons within a minicolumn share the same proximal basal segment. On a higher level, minicolumns are organized into layers (Fig. 1c). And within a layer, minicolumns share the same feedforward input, although they can have different receptive fields.

A dendritic segment activation can cause different effects on the neural cell depending on the segment’s type. A proximal basal segment activates its neuron, while the other two types of dendritic segments play a modulatory function. A proximal basal segment is activated if a number of active proximal inputs within its receptive field exceeds a dynamic threshold. This threshold is determined by a “k-winners take all” rule within a layer. That is, final activity of proximal basal segments, and therefore neurons in a layer, depends on their relative ability to match an incoming feedforward spatial pattern.

On the other hand, distal basal and apical dendritic segments become activated if the number of their active inputs exceeds a fixed threshold. Distal basal segments grows synapses to the cells within a layer, while apical dendritic segments grow synapses to the cells in other layers. Their cumulative modulatory function is to affect the cell’s activation priority within a minicolumn. When these segments become activated, they switch the neuron into the so-called predicted state.

A neuron in the predicted state means that it is expected to become activated with the next feedforward input pattern. Note that since neurons in a minicolumn share the same proximal basal segment, its activation should lead to the activation of the entire minicolumn. However, a neuron in the predicted state inhibits the activity of all non-predicted neurons within a minicolumn preventing their activation. A predicted neuron has the priority because it matches both the spatial feedforward input and the spatial–temporal activity context captured by its modulatory segments. Hence, if the prediction comes true, the activated cell in a minicolumn is an exact representation of this captured spatial–temporal context. As a result, active minicolumns within a layer represent current spatial state, while active neurons within minicolumns represent the current spatial–temporal state.

In our work, we use two HTM framework algorithms: Spatial Pooler and Temporal Memory. Spatial Pooler (SP) [18] is a neural network algorithm that is able to encode dense binary patterns into Sparse Distributed Representations (SDRs) using a Hebbian-like unsupervised learning method. The primary role of SP is to enable feedforward spatial pattern matching specialization of the proximal basal segments in minicolumns within a layer, while the other algorithm—Temporal Memory (TM)—represents a model of the pyramidal neurons cortical layer [17]. It is capable of sequence learning due to the ability of pyramidal neurons to guess future feedforward input by matching the spatial–temporal context with modulatory segments.

Another core feature of the HTM is an extensive use of sparse distributed representations. Such choice is supported by the empirical evidence that cortical representations are both sparse, i.e., only a small percentage of neurons is active at any moment, and distributed, i.e., the information is encoded not with a single neuron but across a set of active neurons [31, 36, 65]. An SDR has a number of useful properties [1].

First, sparse representations are more computationally efficient than dense representations. Also, sparsity leads to higher specialization of neurons as they fire much more selectively. The distributed aspect complements it with high noise robustness because in a high-dimensional space, there is an extremely small chance that two random SDR vectors have a significant overlap; in the vast majority of cases, they are expected to be exactly or near orthogonal. From a set-theoretic viewpoint, we can treat SDR vectors as sets with *OR* as a union operation and *AND* as an intersection operation. Additionally, a dot product may act as a semantically meaningful measure of similarity between SDR vectors. As a result, SDRs have a dual nature. On the one hand, SDRs represent discrete objects or symbols because they rarely overlap in most cases. On the other hand, there is continuity—for example, in the vicinity of an object’s SDR or when two objects share common features that contribute to their similarity.

There are several key aspects that differentiate HTM framework neural networks from Artificial Neural Networks (ANN) and make it a more biologically plausible model.

First of all, neurons communicate with discretized binary spikes rather than real-valued data. Secondly, it works with sparse distributed representations. Thirdly, besides feedforward neural connections, it defines modulatory connections. Lastly, it uses Hebbian-like learning at its core instead of backpropagation.

### Cortico–basal ganglia–thalamocortical circuit

**Fig. 2:**
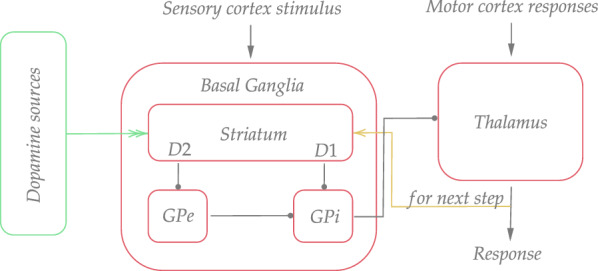
The scheme of the selection circuit. Blocks represent corresponding biological objects: GPi—the globus pallidus internal segment; GPe—the globus pallidus external segment; D1, D2—the dopamine receptors of striatal projection neurons; triangle arrows—excitatory connections; circle arrows—inhibitory connections; double triangle arrow—dopamine connections

To make an agent’s behavior more biologically plausible, we were inspired by natural architecture of the brain selection circuit. Among the many loops and circuits in the brain, there is the cortico–basal ganglia–thalamocortical loop, which realizes selection between cortical suggestions. In this paper, we use the basal ganglia–thalamus system (BGT) such as depicted in Fig. 2.

The BGT system selects elementary and abstract actions under some sensory input context. In the BGT loop, the cortex operates with sensory and motor representations. It passes signals to the basal ganglia and thalamus. The basal ganglia (BG) are the most active part of the brain that regulates movement and behavioral aspects of motivation. This is the place where intrinsic and extrinsic motivation are processed. In terms of artificial intelligence (AI), they realize reinforcement learning. In our simplified model, they consist of the striatum and globus pallidus internal (GPi) and external (GPe). The striatum receives signals from the cortex and dopamine sources. Also, it receives delayed thalamic activity. The thalamus, under the basal ganglia modulation, rejects or accepts cortical suggestions. Accepted suggestions are sent back to the cortex.

In the basal ganglia, mainly two types of neurons with receptors D1 and D2 receive signals from the cortex. The D1 neurons are active selectors because they directly reduce the GPi’s tonic inhibition of the thalamus and cause the disinhibition of the selected movement. The D2 neurons, on the other hand, act on the GPi indirectly via the GPe mediator. As a result, a signal coming from the GPi to the thalamus is subjected to dual control.

The dopamine release system (reward and punishment) is responsible for learning in the basal ganglia and, as a result, determines the effect of the BG on the stimulus. Dopamine solidifies the connections between the cortex and the striatum. In response to positive signals, it strengthens D1 receptors while weakening D2 receptors, and in response to negative signals, it has the opposite effect. Whether the signal is positive or negative is determined by the TD error, which is based on the reward with previous and current striatal activity.

### Empowerment

The intrinsic motivation in our agent is based on empowerment introduced by Klyubin et al. [37]. Empowerment is a utility function that estimates the agent’s capability to influence the environment from a specified state. Therefore, it can highlight key states with increased potential for an agent to explore. Empowerment is a dense function and can counter sparsity of the extrinsic rewards, which is a big problem in RL. Also, this function is stable, which means that for a fixed input state in a stationary environment, it gives an exact, not changing, value.

By definition, empowerment is the information channel capacity between a sequence of actions and an agent state:

4 $$\begin{aligned} \begin{aligned}\epsilon (s_t) &= \max _{p(a_t^n)} \sum _{A^n, S} p(s_{t+n} | a_t^n)p(a_t^n) \log \hat{p}(s_{t+n} | a_t^n); \\\hat{p}(s_{t+n} | a_t^n) &= \frac{p(s_{t+n} | a_t^n)}{\sum _{A^n} p(s_{t+n} | a_t^n)p(a_t^n)}, \end{aligned} \end{aligned}$$

where $s_{t+n}$ is the agent’s state at timestep $t+n$, $a_t^n$ is the sequence of actions that leads from $s_t$ to $s_{t+n}$, $A^n$ is the set of all possible combinations of actions with length *n*, and *S* is the set of all possible states.

For deterministic environments, Eq. 4 can be simplified. First, we expand the logarithm of the fraction under sum:

5 $$\begin{aligned} \begin{aligned} \epsilon (s_t)&= \max _{p(a_t^n)} \left[ {-\sum _{A^n, S} p(s_{t+n} | a_t^n)p(a_t^n)\log p(s_{t+n})} \right.\\ &\left. {\quad + \sum _{A^n, S} p(s_{t+n} | a_t^n)p(a_t^n)\log p(s_{t+n} | a_t^n)}\right] \\&= -\sum _{S} p(s_{t+n}) \log p(s_{t+n}) \\&\quad + \max _{p(a_t^n)} \sum _{A^n, S} p(s_{t+n} | a_t^n)p(a_t^n) \log p(s_{t+n} | a_t^n). \end{aligned} \end{aligned}$$

Then, given that in a deterministic environment, any *n*-step sequence of actions $a_t^n$ only determines a single corresponding trajectory $s_t \rightsquigarrow s_{t+n}$, probability $p(s_{t+n} | a_t^n)$ is either 1 or 0, hence either $p(s_{t+n} | a_t^n)$ or $\log p(s_{t+n} | a_t^n)$ is zero. Therefore, for this case, the second term is zeroed out:

6 $$\begin{aligned} \epsilon (s_t) = -\sum _{S} p(s_{t+n}) \log p(s_{t+n}). \end{aligned}$$

As a result, finding the empowerment value for the state $s_t$ only requires knowing a probability distribution over states $s_{t+n} \in S$ that are reachable from $s_t$ in exactly *n* steps.

## Hierarchical intrinsically motivated agent (HIMA)

The hierarchical intrinsically motivated agent (HIMA) is an algorithm that is intended to exhibit an adaptive goal-directed behavior using neurophysiological models of the neocortex, basal ganglia, and thalamus. This section provides details of the HIMA operation principles. First, we delineate functions of main components from a bird’s eye view and then describe each part in-depth in the following subsections.

We assume that the agent has sensors and actuators enabling it to gain experience through interaction with the environment. We also assume that sensors provide it with enough information to determine the state of the environment, the state of the agent itself, and rewarding behavior. The neocortex model is used to form hierarchical internal representations of raw sensory input and a model of the environment. The basal ganglia model provides an association of internal representations projected from the neocortex with rewarding signals and selects appropriate actions via thalamocortical loops. The Dreaming component models circuits of the brain responsible for the initiation of planning via the model of the environment in the neocortex, improving the learning speed. The Empowerment module is in charge of producing intrinsic motivation signal utilizing the environmental model learned by the neocortex to guide exploration to the most promising states first.

**Fig. 3:**
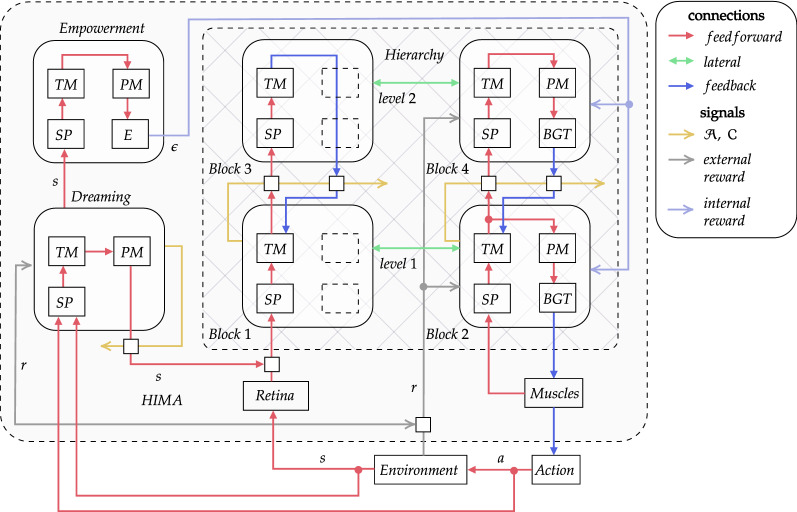
HIMA with hierarchy of two levels and *Block 2* as an output block

The agent’s architecture can be described in terms of blocks (Fig. 3). There are six interconnected blocks. *Block 1, 2, 3*, and *4* are organized into a hierarchy that enables automatic abstract actions formation through exteroceptive (retina) and proprioceptive (muscles) input (see Sect. 4.1). This structure performs the agent’s behavior generating actions representation [sent by muscles to the environment], guided by information from the reward signal.

The reward signal has two components. The first one is the [external] reward corresponding to the vital resources that an agent gets from the environment. The second one is the intrinsic reward generated by the *Empowerment* block, which mainly serves as a motivator for an exploration of the environment (see Sect. 4.3).

The last—*Dreaming*—block is an algorithm that learns a forward model of the environment by receiving the same inputs as the agent and serves as a virtual playground for the fine-tuning of the agent’s skills (see Sect. 4.5). The Dreaming module has an ability to short-circuit the agent–environment interaction loop to mimic operating in imagination. For the duration of the dreaming process, the agent is kept detached from the environment and interacts as usual but only with the Dreaming module instead of the environment.

Each block in turn consists of sub-blocks. Our model has five sub-blocks. The Spatial Pooler (SP) sub-block is an algorithm that forms an internal representation of the input. Temporal Memory (TM) learns sequences of input patterns in an online fashion (see Sect. 3.2). The Basal Ganglia and Thalamus (BGT) sub-block is used for best action selection and reward aggregation (see Sects. 3.3 and 4.2). Pattern Memory (PM) is an algorithm that stores patterns generated by a Spatial Pooler, which are required by other sub-blocks (see Sect. 4.4). Finally, the Empowerment (E) sub-block represents the main computations for the intrinsic reward evaluation of a current state.

### Abstract actions

In this subsection, we describe how abstract actions, or options, arise from the flow of sensory input (to match our approach with Options Framework see Sect. 8.1). We divide the sensory input of the agent into two flows: exteroceptive, or visual (coming from retina), and proprioceptive, or motor (coming from muscles) (Fig. 3). Hence, there are two sub-hierarchies: the visual hierarchy is on the left (*Block 1* and *Block 3*) and the motor is on the right (*Block 2* and *Block 4*).

Consider the visual hierarchy first. An agent’s observation is represented by a binary pattern of retina cells’ activity. This pattern gets into *Block 1*, where it is encoded by SP to form a low-level elementary state $p_t$. Then, the corresponding SDR comes to TM, which learns sequences of elementary states. As discussed earlier in Sect. 3.2, TM considers patterns within the context of the currently observed sequence of patterns—i.e., *Block 1* TM considers states within the context of the agent’s current state trajectory.

Two signals—anomaly ${\mathcal {A}}$ and confidence ${\mathcal {C}}$—indicate the TM state and gate information flow from *Block 1* up to *Block 3*. An anomaly expresses the degree of surprise for an input pattern $p_t$ at this timestep. Confidence, on the other hand, corresponds to how strong the TM prediction for the next pattern is, given the current pattern. If both—the anomaly and confidence—are simultaneously high enough for $p_t$, it means that we could not expect $p_t$, given the previous context, but know in advance what could follow it next, given the current context. The former tells us that the previously observed familiar sequence has ended, while the latter indicates that another familiar sequence has started. In other words, we observe the switch between learned state sub-trajectories. In this case, $p_t$ is selected to represent the started pattern sequence on the next level and $p_t$ is passed to the SP of the *Block 3*. As a result, the learned sequence of elementary states forms a higher-level, or abstract, state, which is represented by its starting pattern $p_t$.

The second-level TM learns sequences of abstract states. The output of *Block 3* is sent back to *Block 1*. It enables us to mark subsequent elementary states as elements of an abstract state (sequence). When the first-level TM cannot predict the next low-level state, i.e., when ${\mathcal {C}} = 0$, denoting the end of the learned state sub-trajectory, then the second-level TM may still predict what the high-level state goes next. Therefore, it can provide the first level with the sequence representative, which is its starting state pattern, via feedback connections. If it has successfully resolved the struggling low-level prediction, then two consequent abstract states can be joined into a single abstract state.

The motor hierarchy performs in the same manner, although it has a special ability to generate behavior. *Block 2* TM learns sequences of low-level muscle activity, or elementary actions, and the second-level TM learns sequences of high-level actions, or abstract actions. Abstract actions are formed and represented in the same way as abstract states. Additionally, the TM output is clustered with the PM sub-block on both levels of the motor hierarchy (see Fig. 3).

An agent’s behavior is generated by the BGT sub-blocks hierarchy. Each BGT sub-block selects one action pattern among the input clusters provided by PM and sends it down the hierarchy via the feedback connections. That is, the first-level BGT selects an elementary action pattern and sends it directly to the Muscles module, which performs a corresponding action in the environment, while the *Block 4* BGT selects among abstract actions. The *Block 2* TM predicts the next elementary actions using the current active action pattern and the feedback sent by *Block 4* BGT. The predicted action has an increased probability to be selected with *Block 2* BGT. In other words, the selected abstract action causes the first-level BGT policy to follow the corresponding sequence of elementary actions with an increased probability. However, the first-level BGT still has a chance to interrupt the selected abstract action in favor of the elementary action with a higher immediate reward. BGT sub-blocks learn to choose better actions (elementary or abstract) through the reward signal (see Sect. 4.2).

Both hierarchies—visual and motor—have reciprocal lateral connections that help disambiguate the visual and motor input. It makes the system to be noise-tolerant. Lateral connections also correspond to the visual hierarchy projections to the BGT sub-block. The BGT uses elementary and abstract states to predict the outcomes of actions, which helps it select the most profitable option given the context.

### Basal ganglia–thalamus (BGT) system

In our model of the basal ganglia–thalamus (BGT) system described in Sect. 3.3, the input signal for the basal ganglia comes from the cortex as a stimulus to the striatum neurons D1 and D2. In HIMA, this cortical input is represented by the signal from the lateral connection of the corresponding block with the visual hierarchy block of the same level (Fig. 3). It is SDR st of size $k_{in}$ with fixed sparsity rate.

The striatum is the central receiver of signals in the basal ganglia and the central evaluator of them. It builds value function for pairs: stimulus, response (*Q* function in RL terminology). This response is a representation of an option for corresponding stimulus. The striatum is the place where the reinforcement learning takes place via dopamine modulation (more details in Sect. 8.2).

In HIMA, there are two causes of the dopamine sources innervation: extrinsic reward $r_t$ and intrinsic motivation signal $\epsilon (s_t)$. We suppose that for each incoming dopamine signal $\rho$ a separate zone of the striatum is working forming two parallel pathways. Processing for both pathways is computationally identical.

The resulting striatum’s output—two vectors $d_1, d_2 \in {\mathbb {R}}^{k_{\text{out}}}$ corresponding to two dopamine receptors—is weighted sum of pathway outputs. Weights are pathway priorities pr, such that $\text{pr}^{\text{int}} + \text{pr}^{\text{ext}} = 1$:

7 $$\begin{aligned} d_\alpha = \eta d_\alpha ^{\text{int}} \text{pr}^{\text{int}} + d_\alpha ^{\text{ext}} \text{pr}^{\text{ext}}, \text { where } \alpha \in \{1, 2\}. \end{aligned}$$

Here, $\eta \in [0, 1]$ is a factor that regulates a scale of an intrinsic signal. We use a simple idea to evaluate priorities: if the agent receives the average extrinsic reward higher than the average minimum extrinsic reward, then it finds the resource well and does not need exploration (high $\text{pr}^{\text{ext}}$). The detailed description of priority formation see in Sect. 8.2. Intrinsic signal priority $\text{pr}^{\text{int}}$ has a crucial role in altering agent’s behavior (we thoroughly discuss it in Sect. 5.4.2).

The striatum outputs characterize value for stimulus–response pairs and are the GPi inputs (see Sect. 3.3). We form basal ganglia output $\text{gpi} \in \mathbb {SDR}(k_{\text{out}})$ in the GPi in several sequential steps. First, we aggregate GPi inputs:

8 $$\begin{aligned} \text{gpi}^{\text{real}} \leftarrow - \text{gpe} - d_1 = d_2 - d_1, \end{aligned}$$

where $\text{gpi}^{\text{real}} \in {\mathbb {R}}^{k_{\text{out}}}$; then this vector is normalized:

9 $$\begin{aligned} \text{gpi}^{\text{real}} \leftarrow \frac{\text{gpi}^{\text{real}} - \min {\text{gpi}^{\text{real}}}}{\max {\text{gpi}^{\text{real}}} - \min {\text{gpi}^{\text{real}}}}. \end{aligned}$$

Finally, we binarize it with the sampling from the Bernoulli distribution using $\text{gpi}^{\text{real}}$ to define the distribution parameter for every dimension:

10 $$\begin{aligned} \text{gpi} \leftarrow \mathrm {Bernoulli}(\text{gpi}^{\text{real}}). \end{aligned}$$

The resulting vector forms an output from the basal ganglia.

Now consider the modulation process. The input signal for the thalamus is the set of responses and their weights $\left\{(\text{res}_i \in \mathbb {SDR}(k_{\text{out}}), w_i \in {\mathbb {R}})\right\}$ from the cortex; these responses are ones that the cortex “thinks” could be the answers for input stimulus st. Weights define the significance of the responses. In HIMA, this cortical input to the thalamic part of the BGT sub-block is provided by the corresponding block’s PM sub-block.

The modulation process aggregate an input to the thalamus $\text{res}_i$ and the output from the basal ganglia gpi. First, we calculate an intersection between the complement of gpi—${\overline{\text{gpi}}}$—and $\text{res}_i$. Then, we evaluate each response: $v_i = w_i |{\overline{\text{gpi}}} \cap \text{res}_i |$. After that, these values are normalized with softmax: $p(\text{res}_i) = e^{\beta v_i}/\sum _i e^{\beta v_i}$ ($\beta$ is the inverse temperature). We treat them as probabilities that define parameters of the categorical distribution. Finally, the response is sampled according to this distribution. It forms the output of the thalamus and of the whole BGT sub-block.

### Intrinsic motivation with empowerment

We use empowerment as an intrinsic motivation signal, which was discussed in Sect. 3.4, reasons of such choice will be discussed in Sect. 5.4.2. To calculate empowerment and generate corresponding intrinsic reward, there is a dedicated block in HIMA called the *Empowerment* block (see Fig. 3). It learns and maintains the model of the environment, which helps to calculate the probability distribution over states in *S* that are reachable after *n* steps starting from the given state $s_t$. The model is represented by the TM sub-block.

The workflow of the module consists of learning and evaluating processes. During its operation in the environment, the agent receives sensory input that is preprocessed by the SP sub-block. The resulting SDRs form the sequence of states $s_t, s_{t+1}, \ldots$. The TM for learning uses pairs $s_t \rightarrow s_{t+1}$ constructed from the general sequence of the agent’s states. So, after this process, the module stores information about all transitions from state to state, which the agent has received.

To evaluate the empowerment value, we use several concepts: superposition, clusterization, and distributed evaluation.

One of the distinguishing features of the Temporal Memory algorithm is its superposition of predicted states. This means that a TM prediction is a union of all possible variants of the next state. On the one hand, a superposition is a useful thing because after *n* prediction steps, we immediately have the superposition of all possible $s_{t+n}$ . But on the other hand, such superposition makes it difficult to evaluate the number of occurrences of a specific state in it and to distinguish different states from each other. The structure of TM allows solving the former, while a PM sub-block is used for the latter.

**Fig. 4:**
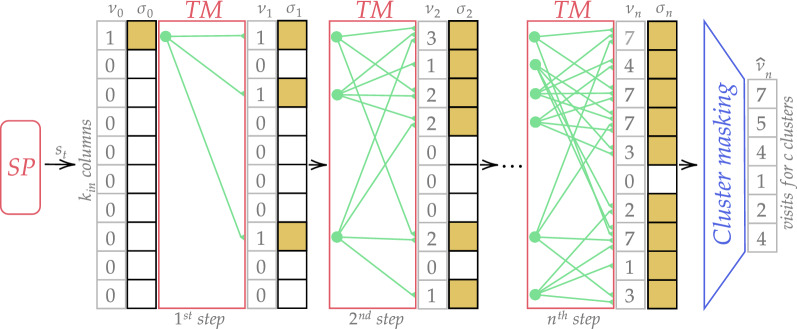
The scheme of visit statistics evaluation. After *n* prediction TM steps the vector of column visits $\nu _n$ is received. This vector is masked by clusters representations that gives ${\hat{\nu }}_n$. This vector represents the number of visits for each cluster after *n* steps from $s_t$

As discussed in Sect. 3.2, TM consists of an array of columns. Each column has a fixed number of cells, each of which has their basal distal segments connecting with other cells. When TM makes a prediction, it depolarizes segments that have enough active presynaptic connections with current active cells. Let $\sigma _0 \in \mathbb {SDR}(k_{\text{in}})$ be an initial SDR for state $s_t$ (hereinafter in this subsection, SDRs will be considered in sparse form, i.e., as a set of active bits indices) and $\nu _0 \in {\mathbb {R}}^{k_{\text{in}}}$ is a vector for visit statistics (see Fig. 4).

In the beginning, only a single, starting, pattern is active, and we write it in visit statistics: $\nu _0^j = 1,\text {where } j \in \sigma _0$. Then, TM makes a prediction based on $\sigma _0$; active segments $\Psi _0$ represent predicted cells and columns. The prediction is $\sigma _1^j = I(G_j^0 \notin \emptyset )$, where *I*(.) is an indicator function and $G_j^0 \subset \Psi _0$ is a subset of active segments for a column *j*. For each active segment $\psi \in \Psi _0$, we calculate an average $\Lambda (.)$ (mean, median, or mode). It is applied to the visit statistics of presynaptic columns $\Phi (\psi )$ for this segment $\psi$ to obtain an estimated number of visits of the pattern encoding by this segment: $\Lambda _0(\psi ) = \Lambda (\{\nu _0^j | j \in \Phi (\psi )\}): \Psi \rightarrow {\mathbb {R}}^+$. Then, we can update visit statistics as follows: $\nu _1^j = \sum _{\psi \in G_j^0} \Lambda _0(\psi )$. So, for other steps, all actions are the same. Finally, we have vectors $\nu _n$ storing distributed visit statistics and $\sigma _n$, a superposition of visited states.

The next step is to split all patterns from the superposition and compute visit statistics for each cluster. The details about clusterization will be discussed further in Sect. 4.4. Here clusters will be used as stored representations $f \in F$ of all possible states that the agent saw. As all representations *f* is an SDR, we can describe the process of clusters masking in Fig. 4.

The first step is to keep only clusters from a superposition: ${\overline{F}}=\{f:|\sigma _n \cap f| > \Theta |f \in F\}$, where $\Theta$ is some similarity threshold. The second step is to calculate visit statistics the same way it was done before: ${\hat{\nu }}_n^q = \Lambda (\{\nu _n^j | j \in f\})$, where *q* is the index of cluster *f*.

After the normalization of ${\hat{\nu }}_n$, it can be considered as probability distribution $p = {\hat{\nu }}_n / \Vert {\hat{\nu }}_n\Vert _1$ over states after *n* steps. Using this distribution and Eq. 6, empowerment $\epsilon _{s_t}$ is calculated. It forms an intrinsic reward that is used in the striatum Sect. 4.2.

### Pattern memory

As has been discussed earlier, we need to store states’ representations being seen by the agent. To take into account temporal variability of the SP encoding, representations are combined into clusters. Because of some features of the input visual signal (Sect. 5), we normally can have different states with similar representations, but for the Pattern Memory (PM) module, distinguishing them does not pose a problem. Problems may appear for empowerment evaluation (more details in Sect. 5.2).

First, the PM module stores set *F* of clusters’ representations $f \in F \subset \mathbb {SDR}$. Then, it updates the characteristic of a cluster called density $\chi _f \in {\mathbb {R}}^{k_{\text{in}}}$, ($k_{\text{in}}$ is the dimension of clusters and state representations). A component of the density can be considered as a probability of the corresponding SDR cell belonging to *f*.

**Fig. 5:**
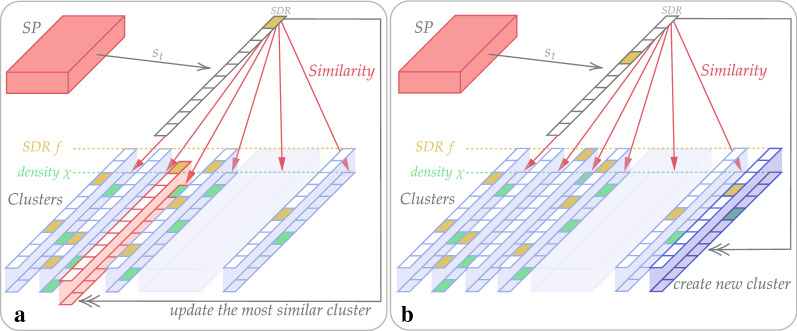
The scheme of the Pattern Memory update process. SP, Spatial Pooler, encodes raw input data to suitable state representation $s_t$ (with dimension $k_{\text{in}}$). Clusters are the set of *c* cluster representation SDRs *f* with their density $\chi _f$. The main idea is based on comparison of $s_t$ and *f* to associate $s_t$ for suitable cluster

The update workflow consists of several steps. Current state representation $s_t$ is compared with each of the clusters (Fig. 5). Here the similarity measure is a scalar product defined as follows:

11 $$\begin{aligned} \text {similarity} (s_t, f) = (s_t, f) = \sum _{j \in s_t} (\chi _f)_j. \end{aligned}$$

If among all clusters, there is a cluster with the highest similarity higher than similarity threshold $(s_t, f_{\text{max}}) > \Theta$, this cluster is updated. Its density is recalculated increasing components corresponding to SDR cells of $s_t$ (Fig. 5A). If the maximal similarity is less than the threshold, a new cluster is created (Fig. 5B).

### Learning in imagination

In HIMA, an agent’s experience is continuously aggregated to build an inner model of the environment. Having it brings the ability to plan ahead and imagine different outcomes—the process that we call dreaming.

Technically, dreaming is a process where an agent’s usual interaction with the environment is short-circuited to the interaction with the inner model of the environment located in the dreaming block. This can greatly support the learning process via learning to solve smaller subtasks in imagination. In practice, dreaming can remedy one of the weakest points of classical RL—sample inefficiency—with a smart, targeted learning rate increase.

An inner model consists of two parts: a transition model and a reward model. A transition model is represented by a temporal memory sub-block that learns state–action transitions $(s_t, a_t) \rightarrow s_{t+1}$. Thus, for a “visited” state–action pair $(s_t, a_t)$, it can predict the next state $s_{t+1}$. A reward model is a learned function over encoded state space. We learn it distributedly, i.e., independently for each state space dimension. A reward estimate for reaching state *s* is an average (median) of reward values corresponding to its pattern active elements: $r(s) = \mathrm {median} \ R(s)$.

Given the potentially overlapping and non-stationary distributed nature of the state–action encodings (due to SP learning in online fashion), we also have to ensure good quality forward predictions. To secure it, we keep track of the learned transition model quality with an additionally learned anomaly model. The anomaly model allows us to evaluate a state prediction miss rate—a prediction anomaly. Like a reward model, an anomaly model is a distributed function, which means it learns a prediction anomaly independently for all dimensions.

We define a state prediction anomaly as an averaged (median) miss rate of its pattern active elements: $\text{an}(s) = \mathrm {median} \ \text{An}(s)$. Because for a deterministic transition $(s_t, a_t) \rightarrow s_{t+1}$, any two parts of this triplet are enough to unambiguously define it, we track the anomaly for tuples $\text{An}(a_t, s_{t+1})$. This way, we can estimate the anomaly for a transition, and we are also able to get the averaged anomaly for state $s_{t+1}$, to which an agent has arrived at the current timestep. A state anomaly helps to decide whether an agent should switch to the dreaming state. To do this, we set a hard anomaly threshold that blocks entering dreaming if the anomaly is too high. Otherwise, we use the anomaly as the probability to switch: $p = (1 - \text{an}(s))^\alpha \cdot p_{\max}$, where $p_{\max}$ is the maximum probability to enter dreaming at zero anomaly and $\alpha$ is a hyperparam to make dependency non-linear. A transition anomaly estimate, on the other hand, is used at each imaginary step during dreaming. It determines whether an agent should stop an early current rollout if it is not certain enough of the next state prediction, i.e., the transition anomaly estimate is over the threshold.

The dreaming block learns during the periods of the agent’s awake activity. At each timestep *t*, it updates its pattern memory and transition and reward models, accepting new information from the environment—current reward $r_t$ and state $s_t$. The anomaly model is updated too, with the transition prediction anomaly $A(a_{t-1}, s_t)$ that it gets from the transition model.

At each timestep, an agent decides whether it will switch to the dreaming state. If so, the dreaming block takes control of the agent by short-circuiting the interaction with it—from now on, the agent acts only in imagination, but not in the environment.

The dreaming process is split into a sequence of independent imaginary trajectories, which we call rollouts [to align with the terminology in the existing RL literature]. Every rollout starts from current real state $s_t$. Thus, the agent is provided with the current observation and reward $s_t, r_t$ and takes action $a_t$. Given a pair $s_t, a_t$, the transition model can make prediction $s^{\text{im}}_{t+1}$ on which state pattern comes next. A reward is calculated directly from the predicted next state: $r^{\text{im}}_{t+1} = R(s^{\text{im}}_{t+1})$. These two pieces form the necessary information to support the next dreamer–agent interaction step $t+1$. The rollout ends when the transition model cannot predict the next state, i.e., the predicted pattern is empty, or when the maximum number of steps is accomplished.

During the dreaming state, the predicted next pattern may be incomplete, or, due to online learning of corresponding spatial poolers, it may even relate to the state in already stale encoding. Basically, we can still just proceed with this prediction as is by taking this pattern as the next state: $s^{\text{im}}_{t+1} := s^p_{t+1}$—if the predicted next state contains garbage, there is a much higher chance the transition memory will predict nothing the step after. However, to help keep the sequence of states in imaginary rollout $\{s^{\text{im}}_j\}$ saner, we use the learned pattern memory for pattern completion. If the predicted pattern is recognized, we correct it with the corresponding cluster pattern. We also check the imaginary transition anomaly, and if it is too high, the rollout is stopped early.

## Experiments and results

A broad range of maze tasks were used in animal-based neurobehavioral research [63] to study spatial working and reference memory [45, 62], search strategies [10, 47], and spatial pattern learning [11]. Similar maze tasks, conducted in simulation, have been proposed and adopted to study corresponding behavioral properties of RL methods [7, 14, 16]. Grid world environments are two-dimensional discrete versions of such mazes. Among their advantages are lower difficulty starting point and slower scaling. They are also much less demanding to the computational resources and do not require highly developed agent’s perception and motor systems. Nevertheless, grid worlds can provide rich and challenging tasks [15, 51, 60].

In our experiments, we studied the following aspects of the proposed model: spatial–temporal pattern representation learning, discovery and usage of state–action abstractions, intrinsically motivated exploratory strategy and learning in imagination through planning. Despite their simplicity, constructed grid world tasks are able to highlight all aforementioned aspects. For example, each task has different states that are visually similar, thus, in order to succeed, it is necessary to learn a helpful representation of states to distinguish and cluster them. Also, part of the experiments were conducted in a four-room environment, which is divided into several zones interconnected with narrow passages. This makes it hard for the agent to switch zones and can be partly mitigated by the use of state–action abstractions or smart exploratory strategy. Finally, the overall difficulty level of four rooms task was calibrated in a way that there was enough room for improvement to justify dreaming capability to speed up the course of agent’s learning. Given that, we treat our decision to test our model in grid world environments as balanced choice between simplicity and experimental depth.

**Fig. 6:**
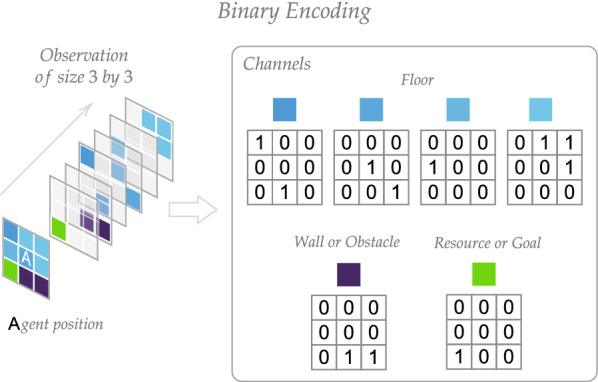
An example of observation and its binary representation. Observation has several channels. Each channel is represented by a binary mask for positions of corresponding objects

Consider a grid world environment. Each its state can be defined by an agent’s position; thus, state space *S* contains all possible agent positions. The environment’s transition function is deterministic. The action space is made up of four actions *A* that move the agent to each adjacent grid cell: up, down, left, and right. However, when the agent attempts to move into a maze wall, the position of the agent remains unchanged. It is assumed that the maze is surrounded by obstacles, making it impossible for an agent to move outside. At each timestep, an agent receives an observation—a binary image of a small square window encircling it. The image consists of several channels. Each channel is a binary mask representing the object positions of the corresponding type in the observation window. There are several channels for floors of different types, one channel for obstacles, and a channel for the vital resource (Fig. 6). The resource position corresponds to a goal state $s_\text{g} \in S$. An agent is positively reinforced with the reward $r = 1$ when it reaches the goal state. On the other hand, at each timestep, an agent receives a small negative reward signal $r(s, a) {:}{=}-\text{cost}(a), a \in A$, where cost(*a*) is a real-valued function that represents an energy cost for every action. We divide the interaction between agent and environment into episodes. At the start of the episode, the agent’s position is initialized from the set of initial states $S_{\text{ini}} \subset S$, and the episode finishes when an agent gets to the goal state or the time limit is reached.

A single test trial lasts for several episodes. As a metric of an agent’s performance during an episode, we use a number of steps required for an agent to reach the goal. An agent is allowed to accumulate experience for the entire duration of a test trial. However, depending on the experimental setup we may also divide a single test trial into a sequence of tasks lasting for several episodes with each task representing an individual set of initial states $S_{\text{ini}}$ and a goal state $s_\text{g}$. For every agent and environment setting, we perform several independent trials with different seed values.

In the following subsections, we describe and discuss the experiments intended to investigate the advantages and caveats of different HIMA modules on their own through performance in relatively simple cases and then in multitasking environments of increasing difficulty. The final experiment is carried out with the full-featured HIMA.

### Abstract vs. elementary actions

The tests represented in this section were designed to compare the performance of an agent using elementary actions only and an agent also using abstract actions in different environment settings. The agent that forms abstract actions corresponds to the HIMA model, but without the Dreaming and Empowerment blocks. The elementary actions agent is the same model but without the second level of the Hierarchy.

#### Four corridors experiment

Tests were conducted on a radial arm maze representing four corridors connected at the center (Fig. 8a). Every episode, an agent starts at the far side of a randomly chosen arm. Initially, a resource is positioned at the center of corridor crossing. Then, after 1000 episodes, the resource is moved to the middle of one of the arms chosen randomly and remains here until the end of the trial, for the next 1000 episodes.

**Fig. 7:**
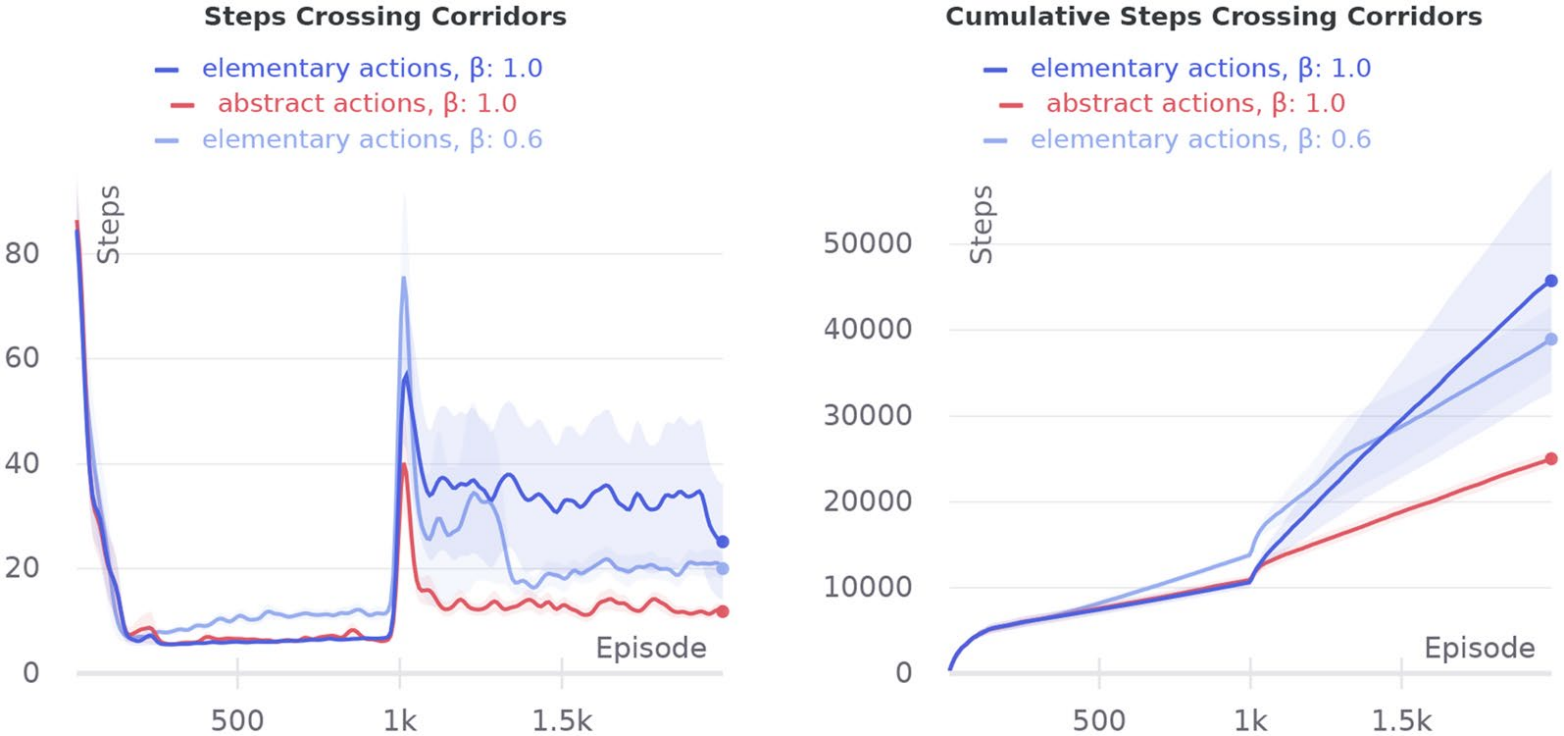
Comparison of agents with abstract and elementary actions on crossing corridors maze

**Fig. 8:**
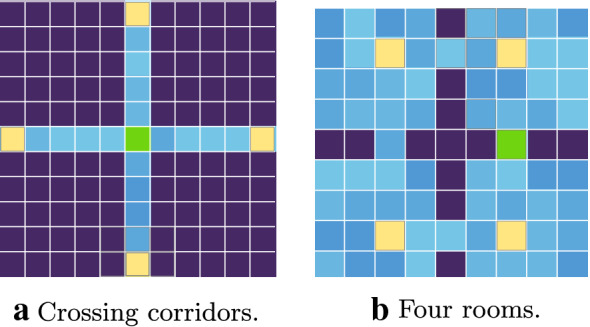
Examples of environments. Yellow—set of initial agent positions. Green—the initial goal position. Dark blue—obstacles. Shades of light blue—floor colors

**Fig. 9:**
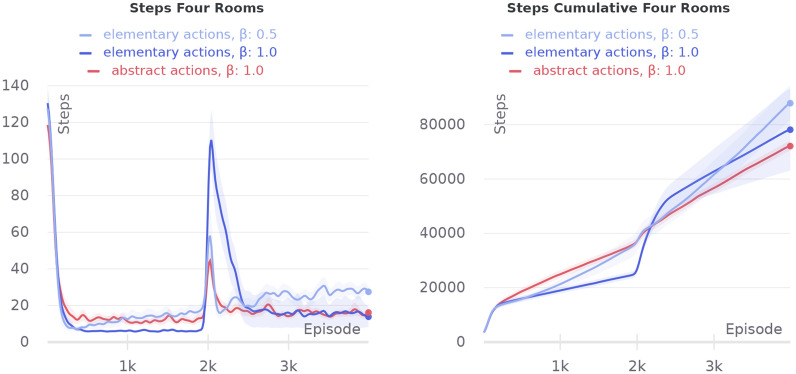
Comparison of agents with abstract and elementary actions on the four-room maze with a restricted set of the initial agent’s positions

As shown in Fig. 7, an agent with abstract actions is much faster to overcome the goal position changing. And as a result, the agent with abstract actions requires fewer steps in total to finish the trial. We also have tried different inverse softmax temperatures $\beta$ for an agent with elementary actions. As we see from the figure, by increasing the temperature, we can improve the elementary actions agent performance but at the expense of optimality at the first half of the trial. The experiment has shown that the agent with abstract actions can explore an environment more directionally than an agent with elementary actions, as the hierarchical structure of the agent allows it to learn four abstract actions for passing each of the corridors. So, when the position of the goal is changed, HIMA has a good chance to get out of the local maximum learned by the first level of the hierarchy.

#### Four rooms experiment

The previous experiment was designed to show the type of cases where our current abstract action model is most effective. However, we also wanted to investigate more common cases in that domain and find out the limitations of our method for the abstract actions formation. So, we have tested our agent in a classical four-room maze, which, because of its bottleneck structure, is often used to test abstract actions.

Trials were carried out on a map having the form of four connected rooms with a resource placed in the left doorway. We consider two variations of the test. In the first one, agent each episode starts randomly in one of the cells from the set marked in Fig. 8b. After 2000 episodes, the resource position is moved to a corner chosen randomly in the left-down room. Another test was performed on the same map, but every episode, the agent starts in any unoccupied randomly chosen cell. The goal state is relocated in the same way after 2000 episodes.

In the first variation of the experiment, the agent’s initial positions were chosen so that HIMA can easily form abstract actions: pass through the door down and right. As can be seen from Fig. 9, the agent with abstract actions performs better after the goal position changing than the agent of elementary actions with the same softmax temperature. Although we can adjust softmax temperature to get similar performance during the reward change, it is still worse than a strategy with abstract actions in the long run. However, HIMA learns the suboptimal trajectory to the goal as can be seen from the first half of the learning curve.

**Fig. 10:**
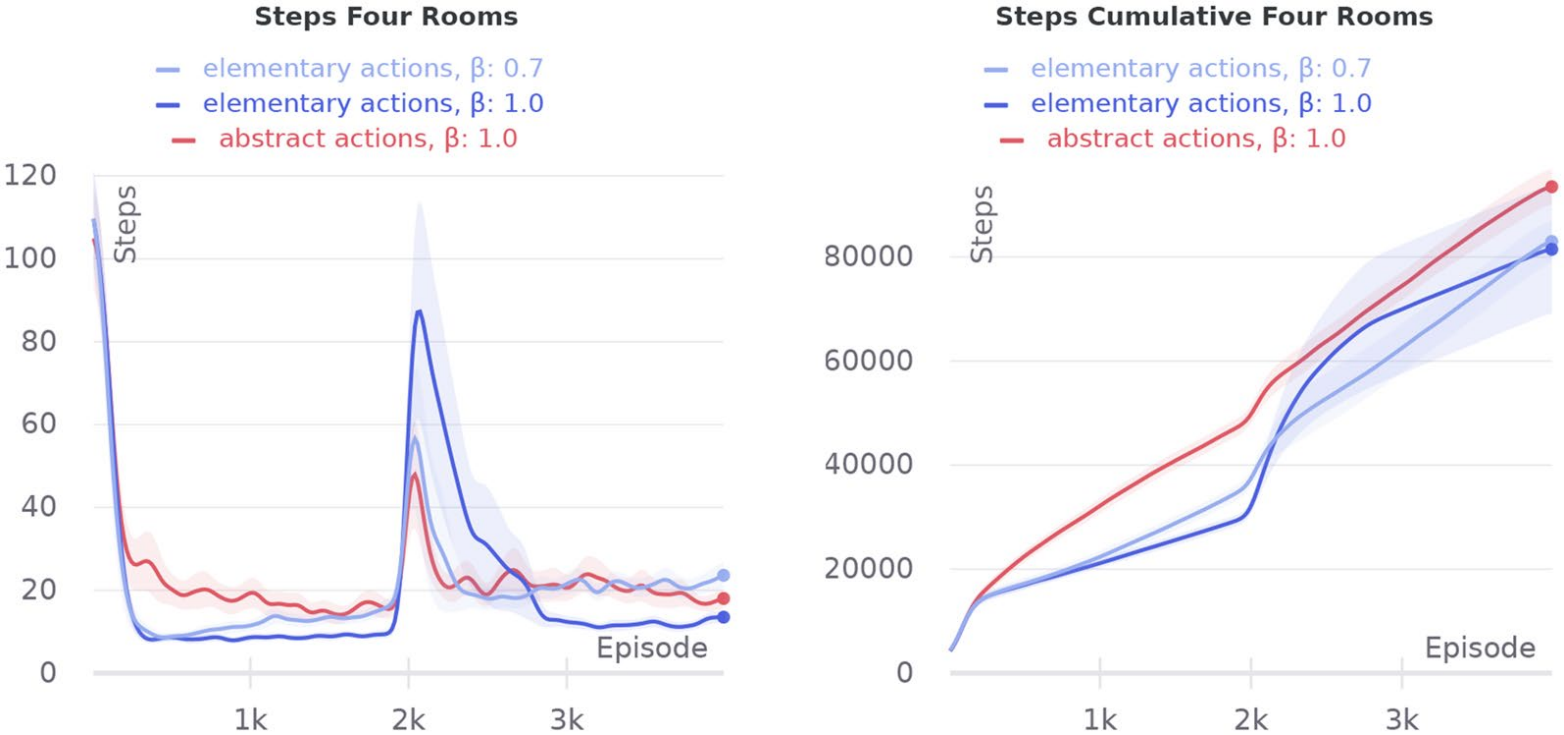
Comparison of agents with abstract and elementary actions on the four-room maze without restrictions on the agent’s initial state set

**Fig. 11:**
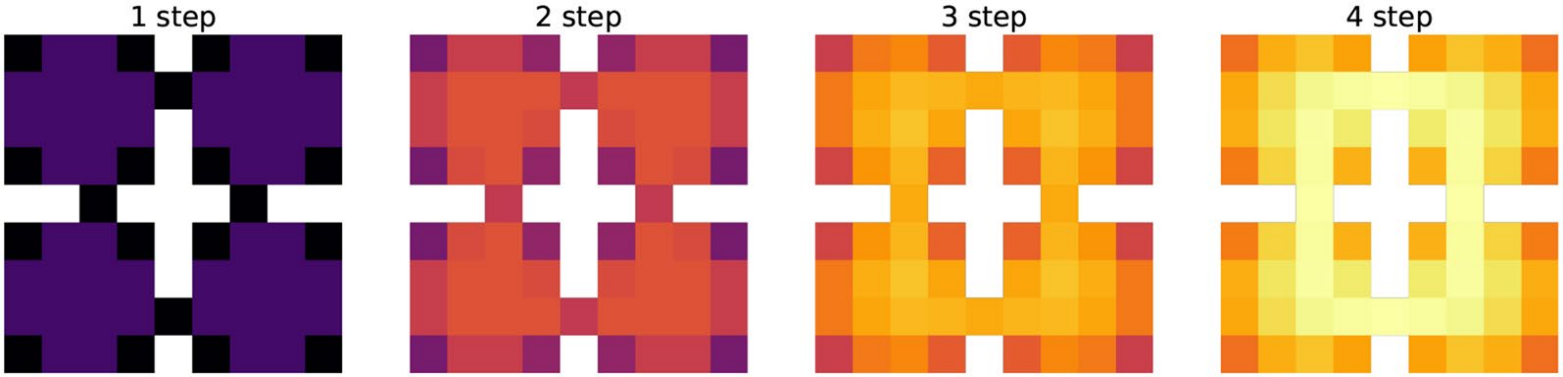
Ideal empowerment fields. All values are in the same limits and can be compared with each other. Darker color—lower value, lighter—higher value. The walls are not shown

**Fig. 12:**
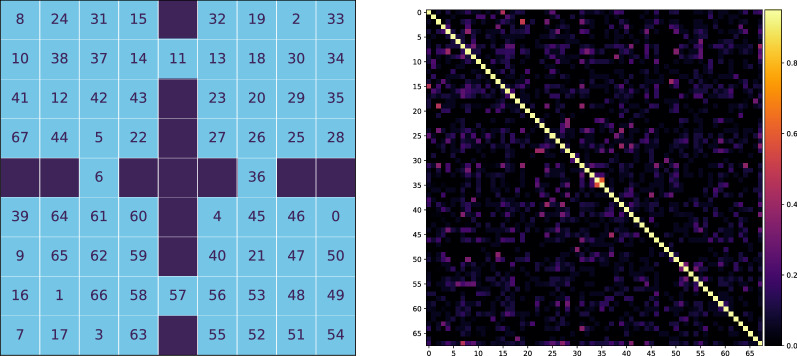
Clusters in four rooms. On the left part is the mapping between the number of cluster and corresponding state in the environment. On the right one is the similarity matrix. The rows and column are the indexes of clusters. The similarity value is shown by color

In the second experiment, there is much more variation between possible trajectories. For now, our HIMA model is not capable to generalize abstract actions by a goal, but it learns the most repetitive action sequences. As long as an agent can start in any position, it is not possible to distinguish the most repetitive action sequences here. So, in such cases, our method does not guarantee to form useful abstract actions. Therefore, as can be seen from Fig. 10, the problem with suboptimality of the abstract actions becomes more vivid. And as long as an agent starts from different positions, directional exploration, which usually helps to pass through bottleneck states, is not so crucial.

The experiments have shown that HIMA is capable of learning useful abstract actions that improve an agent’s exploration abilities in scenarios with non-stationary goal positions in environments with a low connectivity graph of state transitions. Experiments have also demonstrated that better performance can be reached on tasks where any path to the goal on the transition graph can be decomposed into non-trivial sequences of elementary actions, as for crossed corridors and four rooms with restricted spawn set experiments. Otherwise, there is no guarantee that the strategy with abstract actions will be advantageous even considering the best learning conditions.

### Four rooms and empowerment

In this subsection, we evaluate the model of empowerment (Sect. 4.3) on four rooms task. The main goal is to compare empowerment values predicted by our model with the ideal theoretical prediction. In this case the most significant thing is the quality of the transition function, which helps us predict next possible states from the current one. Let us say the ideal empowerment is a value calculated from Eq. 6 having full information about an environment—the final distribution of the reachable states *S*. This case corresponds to having the perfect transition function. On the other hand, the TM empowerment is a value calculated as was described in Sect. 4.3 with the learned Temporal Memory.

To begin with, we analyze the ideal empowerment regarding its depth: the prediction of how many steps it uses. For the four rooms task, this analysis is shown in Fig. 11. This is a field of values for 1–4 step empowerment. If the depth is small, then almost all states are equivalent. Such signal is not very useful, as it does not highlight any special places that we want to find. With increasing the depth, the situation is changing, and for four-step empowerment, the special places are clearly visible. We call this set of points $\epsilon$-ring. Some intuition for the set is that it denotes cells from which the agent can reach the most number of states. If the depth is increased, this set will become clearer, but it is very difficult to make such long predictions (the number of possible path variants increases exponentially). So we opt for the four-step case.

As discussed in Sects. 4.4 and 4.3, the clusters should evaluate empowerment with TM. For the purpose of comparison between the ideal and TM empowerment, we learn TM by a random agent walking 10,000 steps in the environment (after this number of steps, TM does not improve its predictions). During this process, clusters also are created. An example of the learned set of clusters is presented in the left part of Fig. 12. In the similarity matrix (on the right in Fig. 12), we can see that almost all clusters are different, but for some of them, the similarity can near 0.5. The latter is bad for empowerment because similar clusters can interfere, and visit statistics $\nu$ will be mixed (4.3). To partially solve it, we use median or mode as a statistic function $\Lambda$. In addition, similar clusters may lead to the false positive TM predictions—TM can start predicting states that actually cannot be the next ones (the so-called phantoms). Generally, this problem can be solved just by increasing the size of an SDR and decreasing its sparsity, but this requires more resources.

**Fig. 13:**
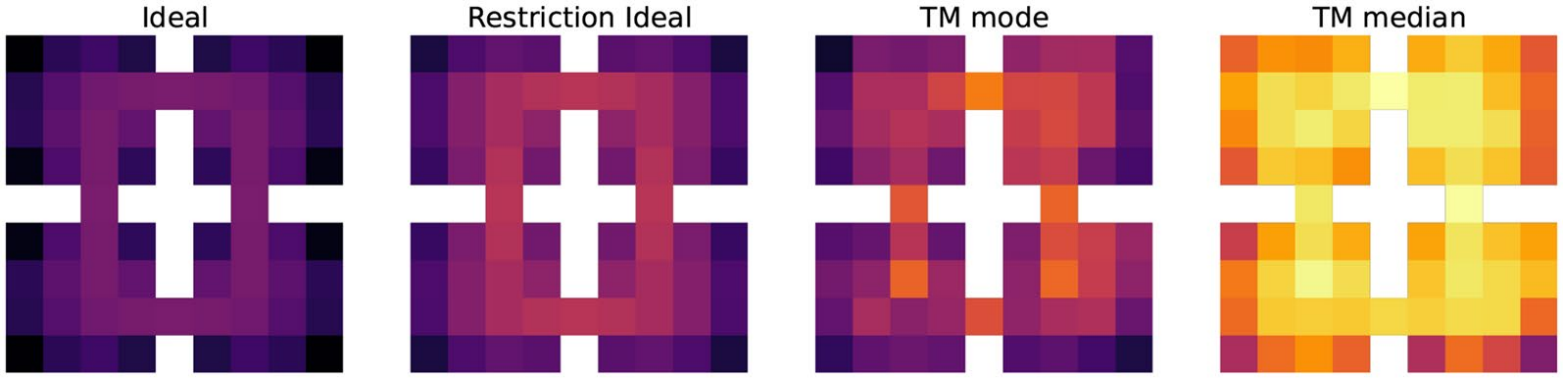
Ideal and estimated empowerment fields in the same value range. Ideal case: uses true transition model. Restriction ideal: the same, but transitions by different actions to the same state are considered as one way. TM mode: TM with mode statistics for visit estimation. TM median: the same with median statistics. Walls are not shown

**Fig. 14:**
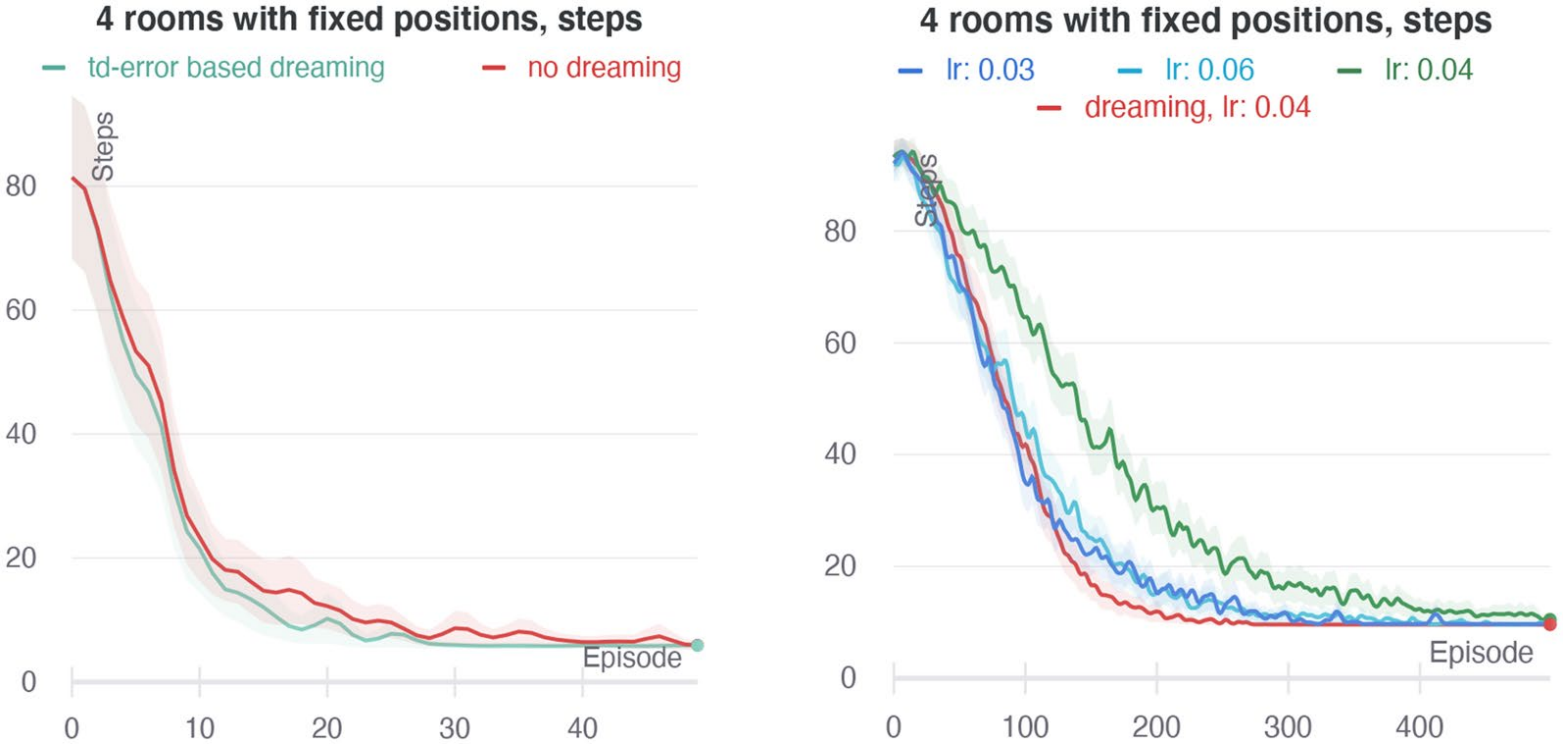
Comparison of different dreaming switching strategies in four rooms with fixed positions experiments. Left: TD error-based switching strategy (green) does not add to performance of the baseline with no dreaming (red). Right: anomaly-based dreaming (red) shows a significant improvement over the baseline with no dreaming (green). It performs similarly to the baseline with the 50% increased learning rate (light blue) and converges twice faster than the baseline with the 25% reduced learning rate (blue, results are *x*2 shrunk along the *X*-axis)

**Fig. 15:**
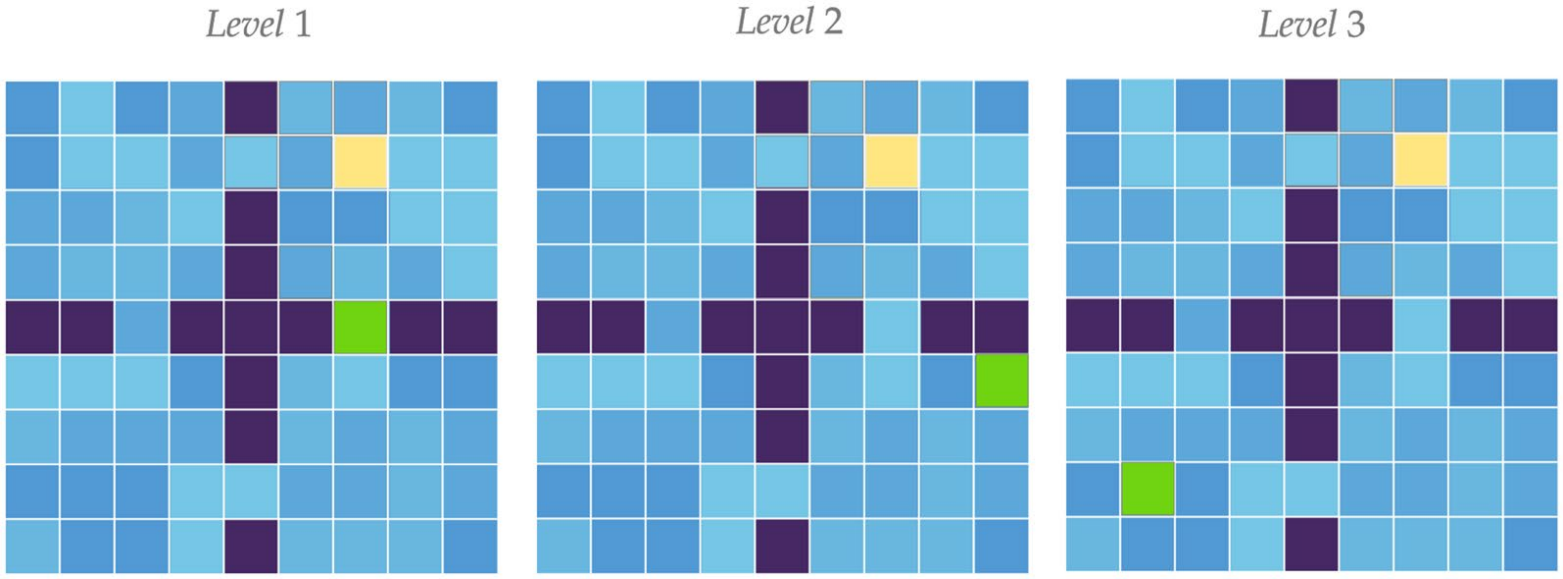
Examples of tasks for different levels. Yellow—the initial agent’s position. Green—initial goal position. Dark blue—obstacles. Shades of light blue—floor colors

The final step of the empowerment analysis is the comparison of the ideal and TM empowerment values. We found that our proposed algorithm for the empowerment estimate cannot handle the case when from a single state different actions lead to itself, which is typical for corner positions. For example, in the top left corner, moving top and moving left both lead to staying in the corner. For this case TM correctly predicts the next state—the corner position itself—but it does not account the number of different transitions $(s, a) \rightarrow s'$, when $s = s'$. One of the possible ways to solve this is to use additional information about actions for TM predictions (like in the dreaming block), but this is a subject for the future research. So for more accurate consideration, we additionally calculate ideal empowerment with this kind of restriction. We also compute empowerment with TM for mode and median statistics. The results are presented in Fig. 13.

We can see that the ideal variant is the least by values compared with others. The TM mode case is the closest to ideal ones, but it overestimates at the gates. The TM median is more overestimated. In our task, overestimation means that prediction is blurred by intersections between states and phantoms (in this case, statistic $\nu$ is shared between states). In both TM cases, $\epsilon$-ring can be distinguished. The main conclusion is that the TM mode can be used as an ideal empowerment approximator. However, we should consider the problem with corners. Heterogeneity of the estimated empowerment value, in our opinion, is the result of both poor semantics in an observation signal and a very basic visual processing system (our model lacks proper visual cortex model, which is also a subject for the future research).

### Dreaming analysis

In this subsection, we discuss experiments with the dreaming. First, we walk through a set of experiments that provided us with the reasoning, which resulted in the final version of the dreaming algorithm described in Sect. 4.5. Finally, we show the effects on the HIMA baseline performance from adding the dreaming block.

During the research and development process of the dreaming algorithm, we were mostly puzzled with two questions. Is the quality of the learned model enough to produce diverse and helpful (correct) planning rollouts? What should the decision-making strategy for starting [or preventing] the dreaming process be?

Above all, we studied pure effects of the dreaming disconnected with HIMA. For that, we took a very basic architecture of an agent instead of HIMA. It had a sequence of SP sub-blocks, which provided a joint state–action encoding. For this encoding, the agent used a classic RL TD-learning method [59] to learn a distributed *Q*-value function, which in turn induced a softmax policy. For such an agent’s architecture, we implemented the dreaming block the same way it is implemented for HIMA. We tested dreaming in four rooms setting where both the initial agent position and resource position were chosen randomly and stayed fixed for the whole duration of the trial. To exclude easy combinations, the trials were selected such that the agent starting position was not in the same room with the resource.

Our initial version of the dreaming switching strategy was to make the probability proportional to the absolute TD error, because a high TD error indicates states where dreaming can contribute the most to the learning process. However, if it is too high, it may also indicate that this state neighborhood has not been properly explored yet; hence, dreaming should not be started as we cannot rely on the inner model. So, we had to find a balanced TD error range, when the dreaming is allowed be activated. Experiments with such strategy showed its ineffectiveness (see Fig. 14 on the left). It has turned out that we cannot rely on the TD error alone to guarantee the local good quality of the learned model.

To get a clue of a better dreaming switching strategy, we decided to investigate situations when the dreaming makes a positive impact on an agent’s performance. Soon enough, a new problem arose—each dreaming rollout can potentially affect further behavior and performance of an agent, so rollouts must be evaluated independently. On the other hand, most of the time, a single rollout effect is negligible or very stochastic. Moreover, independent rollout evaluation does not add to the understanding of their cumulative effect. All of this makes such analysis highly inaccurate and speculative.

In the corresponding experiment, for each trial, we subsequently and independently compared performance of an agent without dreaming with the same agent that dream only once during the learning. So, for each trial, we independently evaluated the outcome of the dreaming for each trajectory’s position of the non-dreaming agent—it showed us all moments where a single dreaming rollout makes a positive or negative impact. The only conclusion we could reach from this experiment was that dreaming more steadily improves performance when it is activated near the starting point. These locations also share lower than average transition model anomaly values. This led us to the final version with anomaly-based dreaming switching.

Tests for anomaly-based dreaming switching were conducted with the same protocol as for TD error-based switching, but on harder tasks. They showed a significant improvement of an agent’s performance. We compared the baseline agent without dreaming and an agent with the anomaly-based dreaming switching strategy (zero-anomaly probability to switch was $p_{\max} = 0.12$). The results are presented in Fig. 14 on the right. Dreaming showed faster convergence to the optimal policy. Based on that, we hypothesized that the effect of dreaming is comparable to the increased learning rate. So, we evaluated the baseline additionally with two different learning rates and included the results in Fig. 14 on the right. The baseline with the 50% increased learning rate (light blue) almost matched the dreaming agent’s performance, while the baseline with the 25% decreased learning rate (blue) was two times slower—it has the number of episodes scaled down two times on the plot for better comparison. Besides the increased speed, we also noted the increased learning stability caused by anomaly-based dreaming.

### Exhaustible resource experiment

Here we investigate how our agent behaves in case resources are exhaustible and their extraction complexity increases. One test trial consists of 30 tasks with three levels of difficulty. There are 10 tasks per level. The maze and an agent’s initial state set is the same as for the four rooms experiment (see Fig. 8b). Tasks of different levels differ by relative positions of the agent and the resource. On the first level, the resource is spawned in one of the two hallways in a room of the agent’s spawn (see Fig. 15). For the second level, the set of the initial resource positions is restricted by two adjacent to the agent’s room. On the final level, the resource can be spawned in any position except the room of the agent’s initial position. A task corresponds to one goal and the agent’s initial positions. The task is changed when the agent visits $s_\text{g}$ more than 100 times, i.e., when the resource is exhausted. The difficulty level of the tasks increases every ten tasks. The trial continues until the agent passes the third level.

In the following subsections, we show the maximum contribution of different features of HIMA to its overall performance on its own. Finally, we carry out the experiment with all the features on and compare our full-featured agent with the baseline. For the baseline, we use the basic version of HIMA with one-level hierarchy and both empowerment and dreaming disabled. The baseline agent uses only one BGT block with one striatum region aggregating the extrinsic reward.

**Fig. 16:**
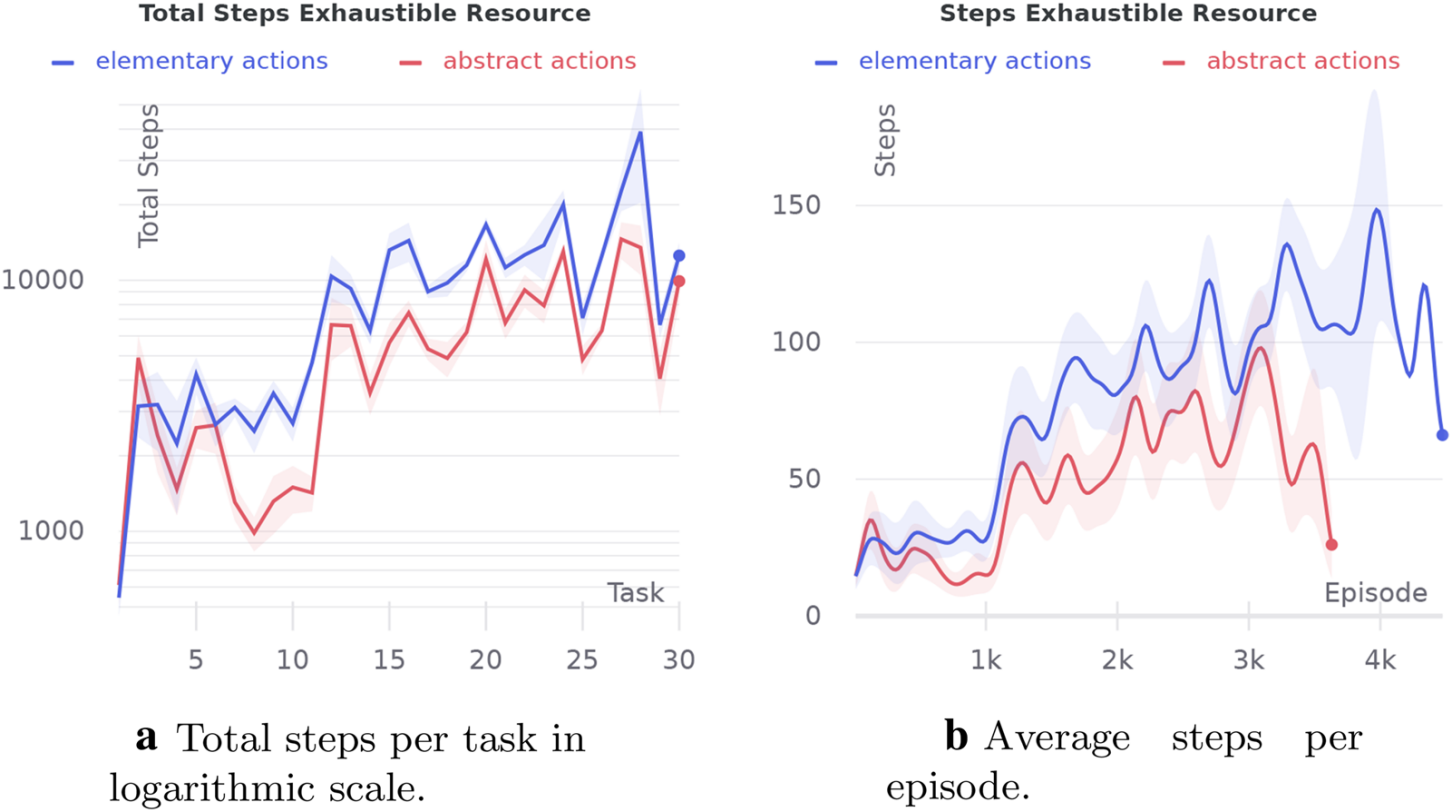
Comparison of agents with abstract and elementary actions in the exhaustible resource experiment

**Fig. 17:**
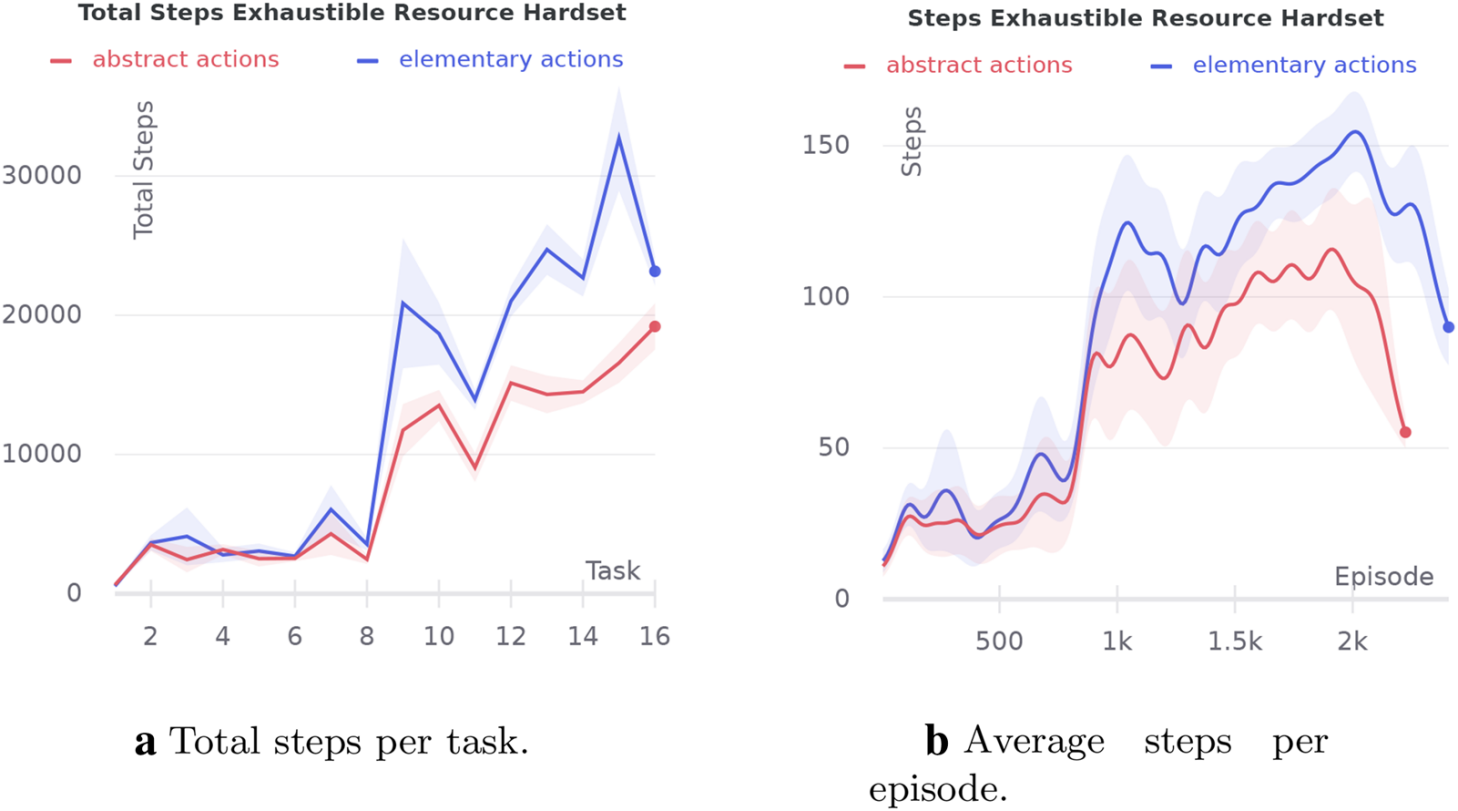
Comparison of agents with abstract and elementary actions in the exhaustible resource experiment on tasks from the hard set

**Fig. 18:**
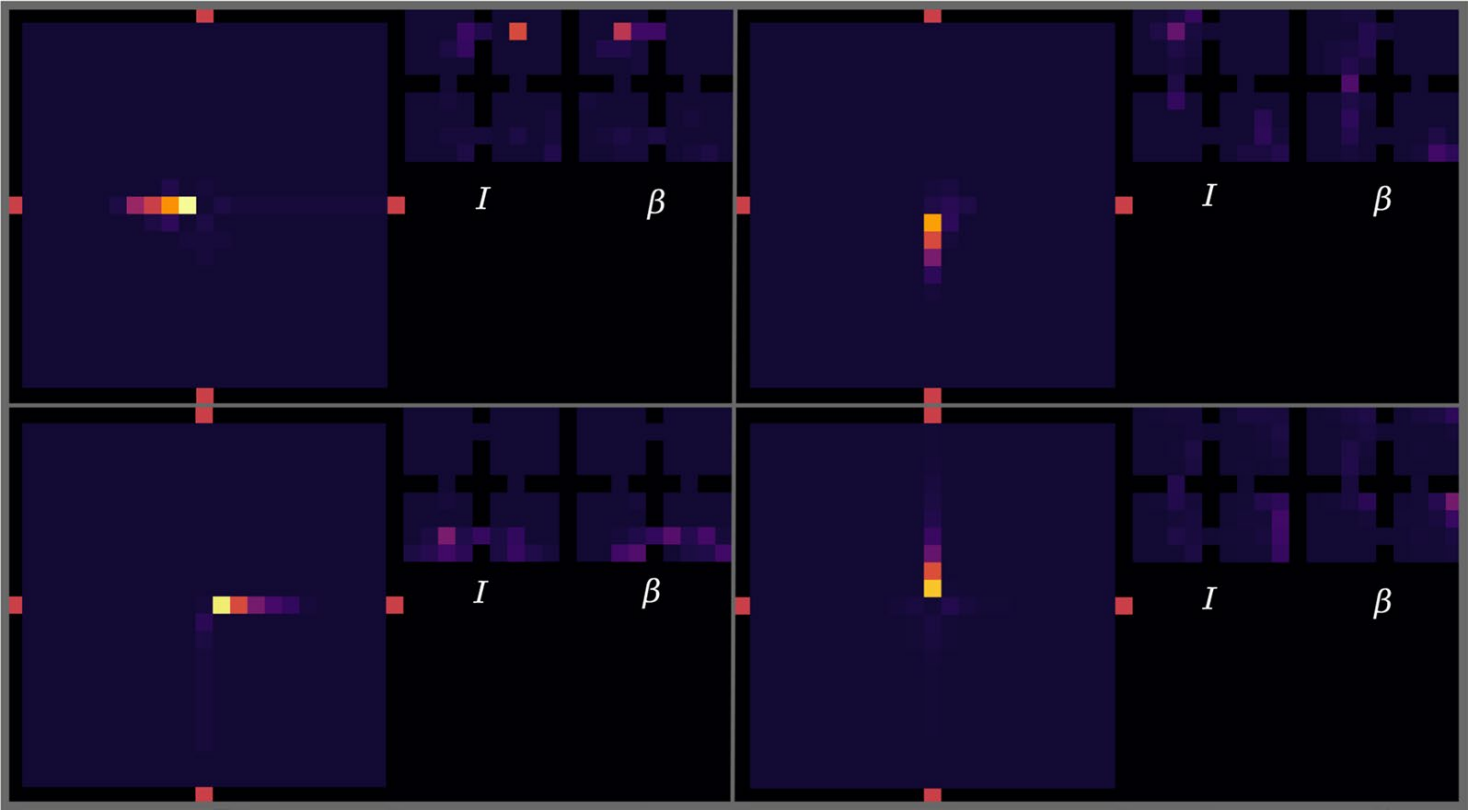
Examples of four options used during the exhaustible resource experiment. The heat map visualizes a number of times the transition to a state was predicted during the execution of the corresponding option. Two small heat maps for each option: *I* is a probability to initialize an option in the corresponding state and $\beta$—terminate probability

**Fig. 19:**
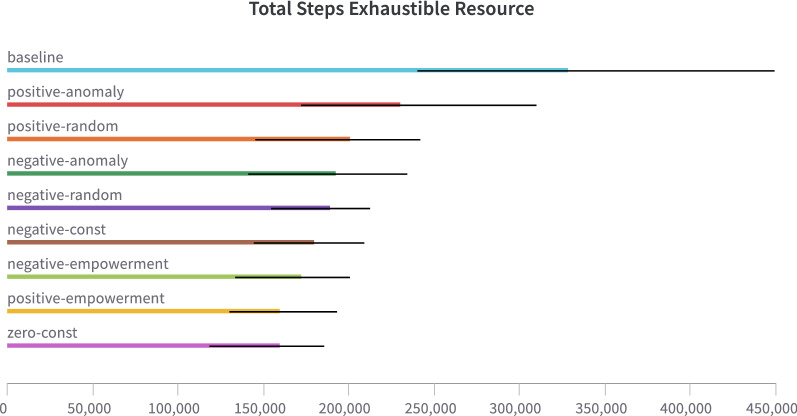
Comparison of agents with different intrinsic modulation signals at Exhaustible resource task. Baseline—the agent without any intrinsic modulation. Agents with prefix “positive” have intrinsic signal from [0, 1] interval. Agents with prefix “negative”—from $[-1, 0]$ interval. The anomaly is simple prediction error for TM. Random is value from uniform distribution. Empowerment is the ideal four-step empowerment

**Fig. 20:**
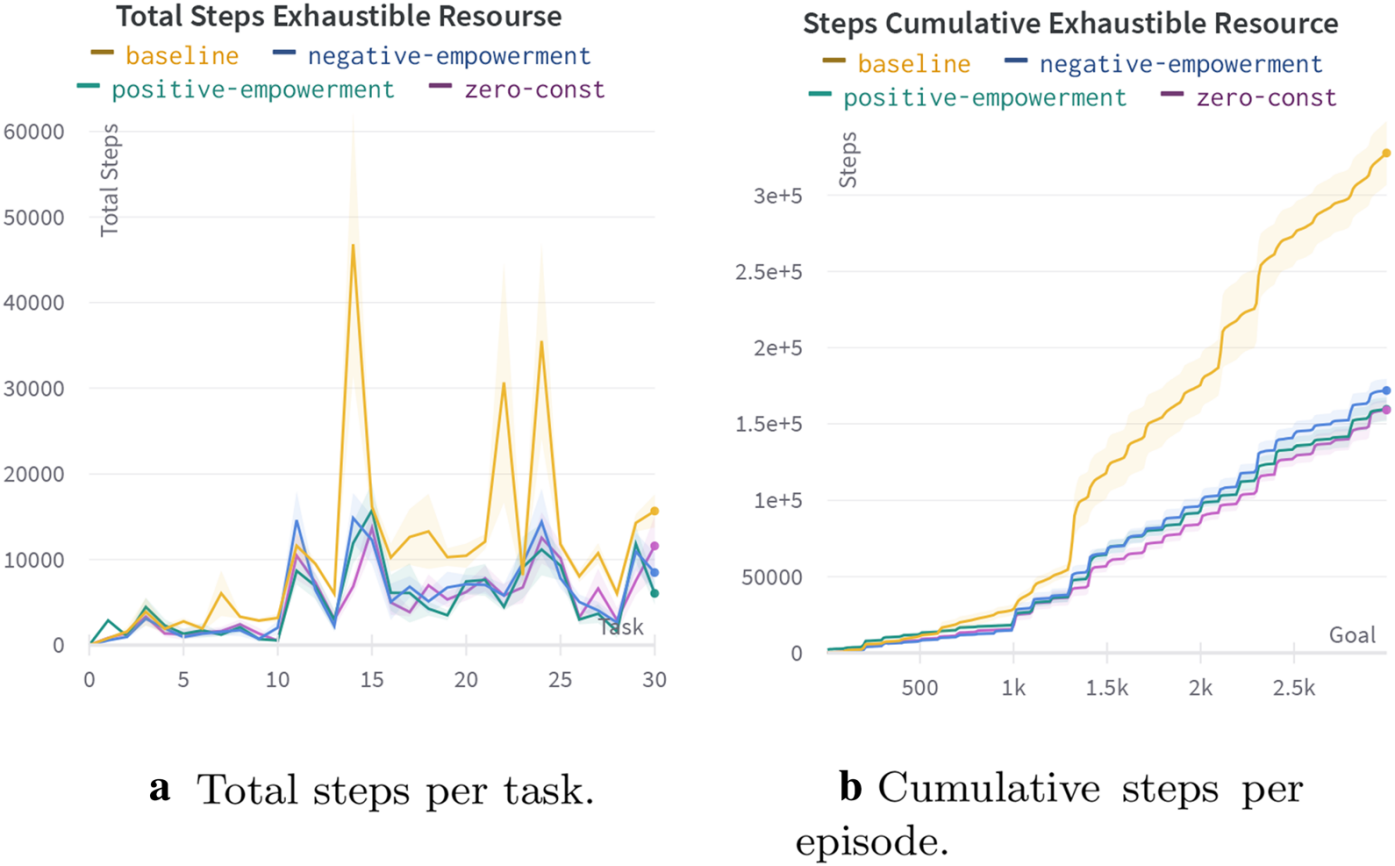
Comparison of agents with a different intrinsic modulation setting in the exhaustible resource experiment. Baseline—the agent without any intrinsic modulation. Negative-empowerment—the agent with intrinsic modulation, where the ideal four-step empowerment is the intrinsic reward (values are shifted to $[-1, 0]$). Positive-empowerment—the same, but the intrinsic reward is shifted to [0, 1]. Zero-const—the same, but the intrinsic reward equals zero

**Fig. 21:**
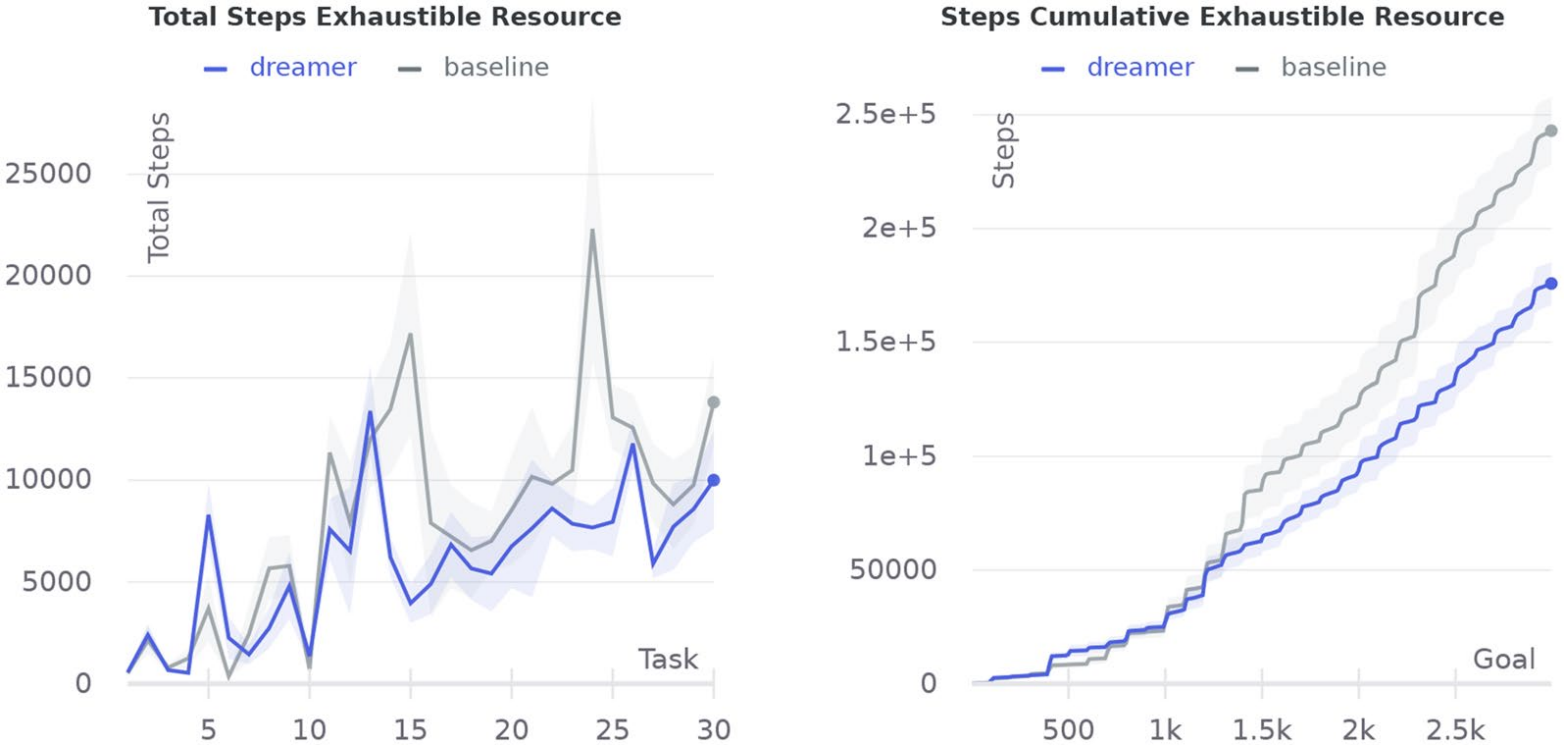
Comparison of an agent with dreaming enabled (dreamer) against the baseline without dreaming in the exhaustible resource experiment

**Fig. 22:**
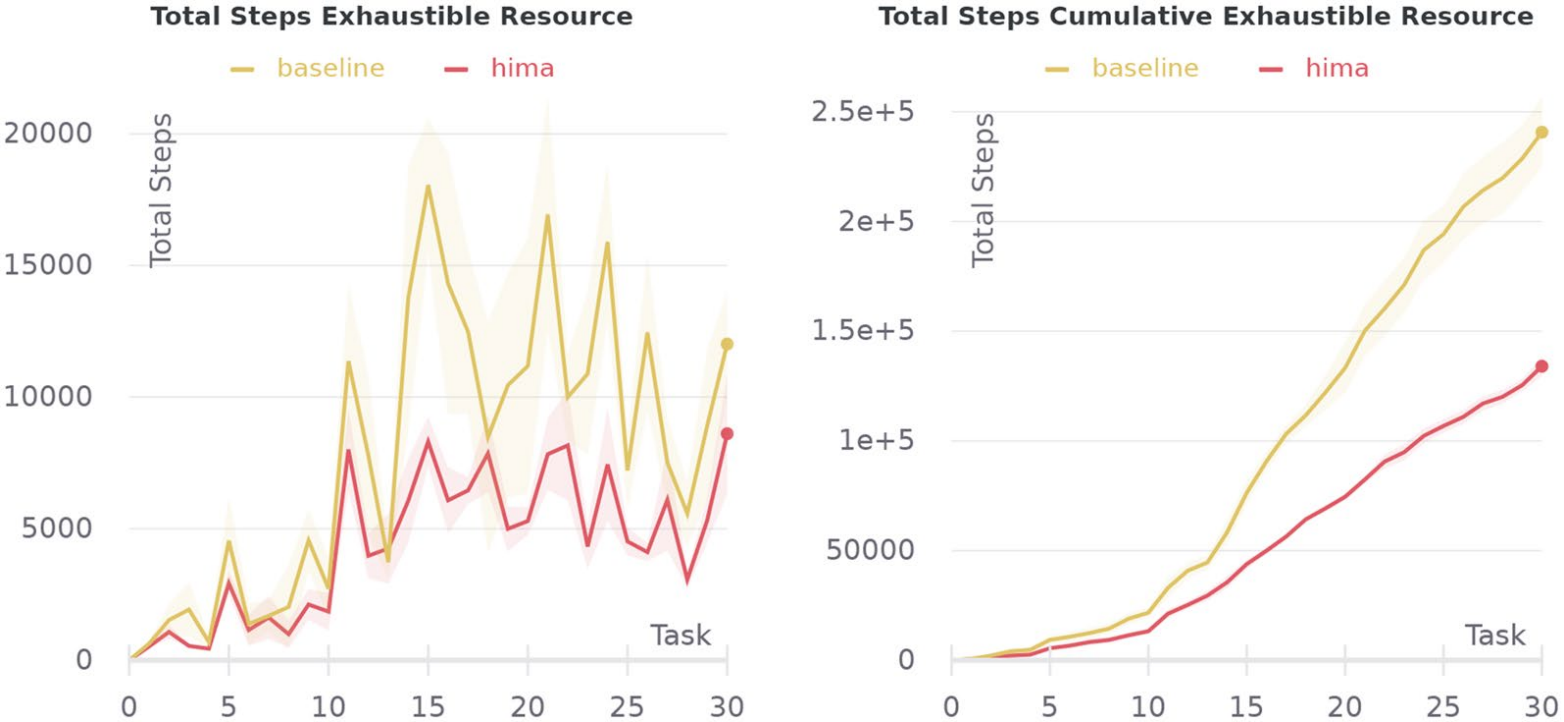
Comparison of full-featured HIMA with a BGT only baseline

**Fig. 23:**
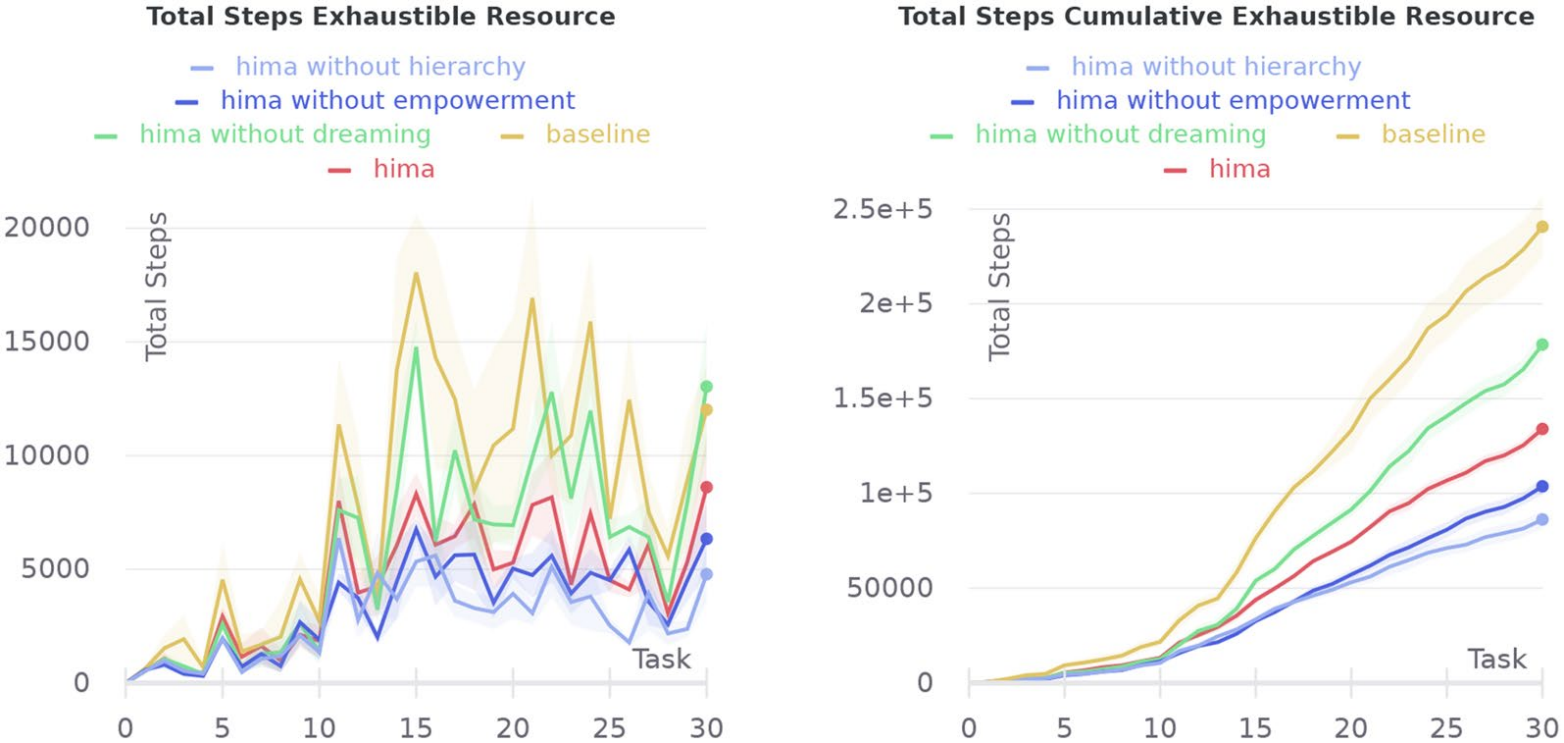
Comparison of a full-featured HIMA with a BGT only baseline and with HIMA without one of the components in the exhaustible resource experiment

**Fig. 24:**
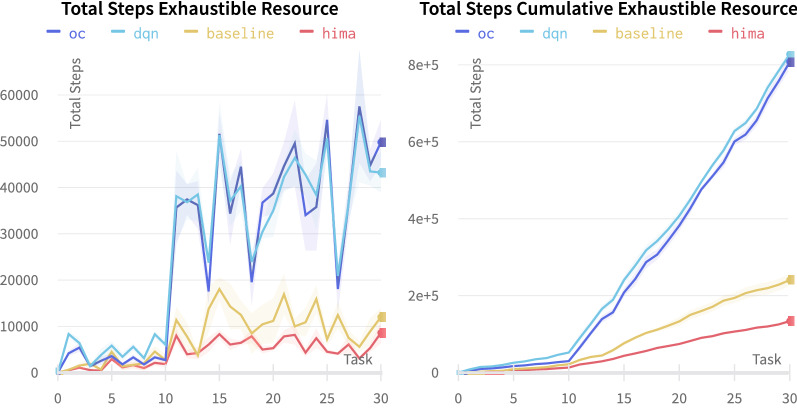
Comparison of baseline (yellow) and full-featured (red) HIMA with DeepRL baselines: DQN (light blue) and Option-Critic (blue)

#### Abstract actions

Here we investigate the effect of enabling the second level of the hierarchy to the baseline HIMA. As can be seen from Fig. 16, the agent with two levels of the hierarchy performs better on average during the tasks. We also have selected a sequence of tasks that consists of conflict situations only and have called it the hard set. In a conflict situation, a strategy learned for a previous task will interfere with the successful accomplishment of the current task. There are eight tasks of the first level and four tasks of the second and the third levels. From Fig. 17, we can see that the agent with abstract actions performs significantly better than the agent with elementary actions. It can also be also noted from Fig. 17a that the difference between the agents arises at tasks of levels two and three, where transitions between the rooms play a crucial role and abstract actions have been learned by the agent already.

There are four examples of abstract actions used during the experiment in Fig. 18, where $I: S \mapsto [0, 1]$ is a probability to initialize an option in a corresponding state and $\beta : S \mapsto [0, 1]$ is the terminate probability. A big heat map for every option visualizes the number of times the transition to a state was predicted during the execution of the corresponding option. Two small heat maps correspond to *I* and $\beta$ functions.

#### Empowerment and other signals

Here we investigate the effect of enabling variants of the intrinsic signal to the baseline HIMA model. We compare the following signals: anomaly, empowerment, constant and random. Anomaly is—a TM characteristic—a percent of active SDR cells that were not predicted ($\text{anomaly}=1-\text{precision}$) for some state $s_t$. Constant is some constant value for all states. Random is some value from uniform distribution (from 0 to 1). Constant and random signals are independent of the agent’s state.

HIMA has its built-in intrinsic motivation. It is caused by an optimistic initialization (see Sect. 8.2). The initial value function is zero, but at every step, the agent gets a small negative reward that is some kind of counter (similar to exploration bonuses), so already visited states will be chosen with less probability (as they will have less value). This feature is always working and helps the agent to start with simple exploration.

To understand the influence of only additional striatum pathway (see Sect. 4.2) we use constant intrinsic signal with zero value (zero-const in Fig. 19). Experiments show a significant improvement in the total steps metric for adding intrinsic pathway in the Exhaustible Resource task. We can conclude that pathways weighting is some kind of “shaker” for an agent. When it reaches resources well it does not use intrinsic pathway (see Sect. 8.2). But then the task is changing (the agent performs badly) and the agent needs more steps to reach a resource, extrinsic pathway turns off—its priority becomes near zero. The agent starts to do random actions (for the zero-const signal) controlled by the intrinsic pathway. This “shakes” the agent’s behavior from stagnation.

We try other signals to make this process more intellectual (Fig. 19). Negative-const is a small negative constant (− 0.01). We assume that this signal strengthens exploration because of optimistic initialization in the intrinsic pathway. But this does not happen, and results become worse than with zero-const.

An anomaly signal can be considered a standard prediction error. Normally, its value is between 0 and 1 (this is positive-anomaly), but also we consider negative-anomaly that is shifted by − 1. In Fig. 19, these signals do not improve the zero-const variant but are better than the baseline.

In Sect. 5.2, we figured that the most suitable depth of the prediction for empowerment signal is four. Our goal is to understand how this intrinsic signal can influence the agent’s performance, so we choose the ideal four-step empowerment signal (that uses the environment transition model) to minimize the negative effects of TM-predicted empowerment (see Sect.  5.2). This signal is shifted to be in [0, 1]—positive-empowerment. And for $[-1, 0]$—negative-empowerment.

We expected that the empowerment signal would help the agent go between the rooms after many failed turns in one room, and this expectation was justified. We found that when the influence of the empowerment signal is big ($\eta \text{pr}^{\text{int}}>> \text{pr}^{\text{ext}}$ see Eq. 7), the agent begins to walk along the $\epsilon$-ring. This can lead to some problems: if the priority of the intrinsic reward is not decreasing, the agent will stay in the vicious circle and not find the resource. Exactly to solve this, we define exponential decay for $\eta$ (Sect. 4.2).

Variants with empowerment show the best performance among other intrinsic signals with semantics. So we can suppose that empowerment is more suitable for our architecture.

From these experiments, we already have made some conclusions. But it needs to pay attention that for all variants of intrinsic motivation their metric one-sigma confident intervals are intersected (Fig. 19). To check that the signal semantics is matter we evaluate agent with positive-random (uniform from [0, 1]) and negative-random (uniform from $[-1, 0]$). As can be seen from Fig. 19 these signals are also among other intrinsic motivation variants. The reason for such behavior can be in the fact that for Exhaustible Resource task priority “shaking” is enough and intellectual intrinsic signals are not necessary.

Also, we perform an analysis of the agent’s work process. In Fig. 20, averaged results of several agent runs are shown. We have found for the steps per each task (Fig. 20a), in some cases, the difference between baseline and others is not so big. As can be seen from Fig. 20, intrinsic motivation signals cannot be distinguished by their performance, but all are better than the baseline without the intrinsic modulation. So we can assume that in this task, the priority modulation (“shaking”) is more important than the exact values of the intrinsic reward.

#### Dreaming

In this subsection, we discuss the effects of enabling the dreaming block to the baseline HIMA. Previously, in Sect. 5.3, we have already shown that dreaming speeds up learning and makes it more stable. Results in the exhaustible resources experimental setup show similar effects caused by dreaming but now applied to the HIMA model (see Fig.  21). In the first-level tasks, dreaming may sometimes decrease performance. However, as the difficulty increases, the positive effects of dreaming grow. Dreaming speeds up convergence during a task. It also accelerates exploration by cutting off less promising pathways.

#### HIMA

So far, we have been considering each component of our agent architecture separately. In this section, we present the results of the tests for the full-featured HIMA model. Before the final experiment, a grid search procedure was performed for several parameters of the agent model with all components enabled. Parameter fine-tuning was carried out on a simplified version of the test with only two first levels of difficulty and five tasks in each one. Then, the HIMA agent with the best parameters has been tested on the full version of the test.

First, we have compared the full-featured HIMA against the baseline HIMA. Figure 22 shows that the full-featured HIMA model performs significantly better than the baseline. They perform on par in the first-level tasks, which do not require transitions between the rooms, and simple softmax-based exploration is enough. The most conspicuous difference between the baseline and HIMA is on the second and third levels, where the abstract actions and the intrinsic reward facilitate more efficient exploration, while dreaming speeds up the whole learning process. The dreaming helps to stabilize the strategy by improving the value function estimate in the striatum.

Second, we have compared the full-featured HIMA against DeepRL baselines: DQN [44] and Option-Critic [5]. The networks for both methods were constructed on top of two fully connected ANN layers. Actor and critic parts of Option-Critic shared network weights and only had separate corresponding network heads. DQN and critic part of the Option-Critic architecture were trained offline, using regular uniformly distributed experience replay. We fine-tuned baselines hyperparameters via grid search on a separate set of seeds within the same testing protocol. Figure 23 shows that both DeepRL methods were unable to adapt to the repeatedly changing tasks and have extremely low performance compared to HIMA.

## Discussion

HIMA has shown an ability to learn an efficient resource searching strategy in tasks with changing goals.

Comparison with DeepRL baselines (Fig. 23) showed that even on simplified grid world environments there are scenarios where DeepRL methods are struggling to effectively find a solution. One reason for this is that in fully connected layers neurons tend to be less specialized compared to neurons in sparse distributed representation. Also, ANN continuous nature do not work well with discrete binary inputs making it hard to converge to local minima with stable representations [25]. Combined, such representation instability and the lack of specialization lead to catastrophic forgetting on task switch, preventing DeepRL methods to accumulate experience.

Additional Option-Critic detailed analysis showed that, for the most part, one option dominates the others or all of them are very short, implying that it tries to solve the task with a high-level policy. This type of degraded behavior is common in Option-Critic architecture and usually indicates an imbalance between the options termination regularizer and the policy over option entropy regularizer. We were unable to achieve such a balance with a hyperparameter grid search for this experiment. The failure of the classic hierarchical DeepRL approach to learn useful sub-policies in scenarios where it was expected to be advantageous additionally justifies our efforts to develop a robust general HRL system.

Additional experiments revealed that not all components of HIMA are orchestrated well for the task, so the component interaction requires further research. Several conclusions can be derived from the results (Fig. 24). The first, and most obvious, is that dreaming has a positive influence on overall performance. Secondly, empowerment and hierarchy have mediocre compatibility with each other. Indeed, all experiments, where the empowerment and hierarchy blocks are enabled simultaneously, yield worse performance than where they are disjoint.

The conflict between empowerment and hierarchy can be explained as a competition of two methods of exploration. Both methods give an agent more directional exploration: the hierarchy with produced abstract actions and the empowerment with the local maxima of its function. However, they are not synchronized well.

First, we found that different components of HIMA share some hyperparameters and their best values for options and empowerment modules are distant. We think that the modulation of driving motivations can be improved. Another reason is that in such small and simple environments both components interfere with each other. Also, the empowerment function is too flat to provide clear and advantageous directions for an exploration in this case. We expect that in more complex experimental setups these problems should became negligible and allow both methods to unleash their potential. For such setup, we suggest robotic experiments, where an agent has much more possibilities to interact and alter the environment. In this case, the empowerment value highlights such interaction possibilities, while abstract actions help an agent to directly explore them resulting in both methods playing along.

## Conclusion

Despite the recent progress of RL in building agents capable of learning complex behavior associated with humans’ and animals’ capabilities, there is no generally accepted framework providing means for effective lifelong open-ended learning so far. Aiming to address this issue in order to build a robust general HRL system capable of continuously learning and reusing acquired skills, we proposed a biologically inspired framework for integrating hierarchical temporal memory, reinforcement learning, and intrinsic motivation, which resulted in a model of an intelligent agent that autonomously acquires knowledge in an environment and then uses it to make better decisions.

Our agent’s hierarchical structure enables it to learn useful spatial–temporal abstractions while also building a compact model of the environment. We use the Temporal Memory model to generate an intrinsic motivation signal called Empowerment, and sparse distributed encoding of states and actions to represent context-dependent states or actions on different levels of the hierarchy. The resulting agent’s behavior is modulated between intrinsically motivated exploration and extrinsically motivated goal-directed behavior. We also enable the agent to reuse the acquired knowledge via dreaming imagination in order to speed up learning.

In our experiments on grid world environments, we demonstrated that the proposed architecture is capable of learning an effective resource-search strategy. We also showed its benefits in the changing tasks scenarios resulting in the faster adaptation. We have compared HIMA to DeepRL methods—DQN and Option-Critic—in such scenarios. Results revealed that even on simplified grid world environments biologically plausible architectures can be advantageous to DeepRL approaches by being more adaptive to changes and less prone to catastrophic forgetting.

In the future, we intend to supplement HIMA with a spatial hierarchy and a biologically plausible visual system capable of semantic feature extraction from a rich sensory input to challenge our architecture in more realistic—robotic—environments. We also see possibilities to improve the abstract action formation algorithm with the incorporation of the explicit goal representation. We expect it to facilitate learning of diverse behavior by an agent. Another promising direction is to supplement HIMA with the grid cells model for better sequence learning and memory anchoring to different environments. Besides, we will further investigate HIMA modules interaction to find better orchestration mechanisms.

## Data Availability

Our model’s implementation source code is publicly available as an archived version and in a repository [39]. It is written in Python, is platform agnostic, and is licensed under the MIT license.

## Appendix

### HIMA and options

In this subsection, we establish a link between our hierarchical model and the Options Framework.

First of all, we should notice that the resulting policy for an option depends not only on a state $s_t$ but also on the previous action $a_{t-1}$: $\pi = \pi (a_t | s_t, a_{t-1})$. However, we can include the previous action as a part of a current state. Thus, we will consider a policy over options $\pi : {\tilde{S}}\times A \rightarrow [0, 1]$, where the state space is defined as ${\tilde{S}} = S \times A$.

The *Block 4* BGT defines the high-level policy—the policy over options $\mu = \mu (o | s)$, while the *Block 2* BGT represents the low-level policy—the policy over actions $\pi = \pi (a | s)$. Both policies are conditioned on the corresponding level current state $s \in {\tilde{S}}$, which is a concatenation of the corresponding block apical and basal inputs.

The selected option *o* forms the *Block 4* output that feedbacks to the *Block 2* to define its policy $\pi$. Every option can be chosen in any state of the environment, i.e., $I = {\tilde{S}}$. The termination condition for an option is determined by ${\mathcal {A}}$ and ${\mathcal {C}}$ signals and by corresponding thresholds in *Block 2*. At timestep *t*, this condition can be determined by state $s \in {\tilde{S}}$, thus, the termination condition depends on time: $\beta = \beta _t(s)$. However, we can get rid of the time component if we stop the TM’s learning.

*Block 2* and *Block 4* gather rewards with a discount factor $\gamma$ until they are reinforced. *Block 2* is reinforced for every action. On the other hand, *Block 4* is reinforced only when the selected option is interrupted or terminated. Therefore, the *Block 4* BGT is reinforced with the reward $R_o = \sum _{t=1}^{m}\gamma ^{t-1} r_t$, where *m* is the duration of an option *o*.

### Reinforcement learning in BGT

The reinforcement learning in BGT consists of two steps: *Q* function evaluation and learning.

At the first step, we evaluate $\text{st}_t$ (names of variables are the same as in 4.2). For this purpose, we maintain two matrices $D^1, D^2 \in {\mathbb {R}}^{k_{\text{out}} \times k_{\text{in}}}$ for each pathway (extrinsic and intrinsic). These matrices are initialized to zero (such initialization is optimistic and realizes some additional exploration similar to count-based variant). Output vectors for each zone of the striatum, $d_1, d_2$, define sparse distributed representation of a state value, which are calculated as follows:

12 $$\begin{aligned} (d_\alpha )_j = \frac{1}{|\text{st}|} \sum _{i \in \text{st}} D^\alpha _{ji}, \text { where } \alpha \in \{1, 2\}. \end{aligned}$$

The resulting output of the whole striatum is calculated using pathway priorities pr, such that $\text{pr}^{\text{int}} + \text{pr}^{\text{ext}} = 1$, to get the weighted values sum:

13 $$\begin{aligned} d_\alpha = \eta d_\alpha ^{\text{int}} \text{pr}^{\text{int}} + d_\alpha ^{\text{ext}} \text{pr}^{\text{ext}}, \text { where } \alpha \in \{1, 2\}. \end{aligned}$$

Here, $\eta \in [0, 1]$ is a factor that regulates a scale of an intrinsic signal. Intrinsic signal priority $\text{pr}^{\text{int}}$ value exponentially decays when $r_{\max} \simeq 0$ and is reinitialized when $r_{\max}$ becomes higher. The priority for the extrinsic signal $\text{pr}^{\text{ext}}$ is defined via reward $r_t$ statistics:

14 $$\begin{aligned} \begin{aligned} \text{pr}^{\text{ext}}&= {\left\{ \begin{array}{ll} 0, &{} \text {if } r_{\max} \simeq 0; \\ \text{clip}\left( \frac{{\overline{r}} - r_{\min}}{r_{\max}}\right) , &{} \text {otherwise.} \end{array}\right. } \quad \\ \text{clip}(x)&= {\left\{ \begin{array}{ll} x, \quad &{} \text {if } x \in [0, 1];\\ 0, &{} \text {otherwise.} \end{array}\right. } \end{aligned} \end{aligned}$$

Here, ${\overline{r}}$ is the reward exponential moving average. The maximum and minimum for reward are also tracked as exponential moving averages:

15 $$\begin{aligned} \begin{aligned} r_{\max}&\leftarrow {\left\{ \begin{array}{ll} r_{\max} \theta _{\max} + {\overline{r}}(1-\theta _{\max}), &{} \,\text {if} {\overline{r}} > r_{\max};\\ r_{\max} \theta ^{\text{dec}}_{\max}, &{} \text {otherwise.} \end{array}\right. }\\ r_{\min}&\leftarrow {\left\{ \begin{array}{ll} r_{\min} \theta _{\min} + {\overline{r}}(1-\theta _{\min}), &{} \text {if } {\overline{r}} < r_{\min};\\ r_{\min} \theta ^{\text{dec}}_{\min}, &{} \text {otherwise.} \end{array}\right. } \end{aligned} \end{aligned}$$

Here, $\theta ^{\text{dec}}_{\min}, \theta ^{\text{dec}}_{\max},\theta _{\min}, \theta _{\max} \in [0, 1]$ are fixed hyperparameters that regulate the rate of averaging and decay.

The second step is matrices $D^1, D^2$ learning. The value function for a pair (st, res) (*Q* function in RL terminology) is represented as a distributed value vector in ${\mathbb {R}}^{|\text{res}|}$, which is calculated as follows:

16 $$\begin{aligned} Q(\text{st}, \text{res})_i = \frac{1}{|\text{st}|}\sum _{j \in \text{st}} D^1_{\text{res}(i),j} - D^2_{\text{res}(i),j}, \end{aligned}$$

where $$i \in \{1, \dots , |\text{res}|\}$$.

At timestep *t*, the TD error (introduced in Sect. 17) is:

17 $$\begin{aligned} \delta ^t_i = \frac{\rho }{|\text{res}|} + \gamma {\tilde{Q}}(\text{st}_t, \text{res}_t) - Q(\text{st}_{t-1}, \text{res}_{t-1})_i, \end{aligned}$$

where $i \in \{1, \dots , |\text{res}|\}, \gamma \in [0, 1]$. Here we use median ${\tilde{Q}}(\text{st}_t, \text{res}_t)$ and normalize input reward signal $\frac{\rho }{|\text{res}|}$, as it results in better convergence. Finally, matrices $D^1, D^2$ are updated:

18 $$\begin{aligned} &D^1_{{\text{res}_{t-1}}(i)j} \leftarrow D^1_{\text{res}_{t-1}(i)j} + {\zeta \delta ^t_i }\\&D^2_{\text{res}_{t-1}(i)j} \leftarrow D^2_{\text{res}_{t-1}(i)j} - \zeta {\delta^t_i}. \end{aligned}$$

Here, $i \in \{1, \dots , |\text{res}|\}, j \in \text{st}_{t-1}$ and $\zeta$ is the learning rate. Note that the elements of matrices that are not considered in 18 are not updated.

## Abbreviations

AI: Artificial intelligence
RL: Reinforcement learning
HRL: Hierarchical reinforcement learning
ANN: Artificial Neural Network
HIMA: Hierarchical intrinsically motivated agent
TD: Temporal difference
IM: Intrinsic motivation
EM: Extrinsic motivation
MDP: Markov decision process
HTM: Hierarchical temporal memory
SP: Spatial pooler
SDR: Sparse distributed representation
TM: Temporal memory
BGT: Basal ganglia–thalamus
BG: Basal ganglia
GPi: Globus pallidus internal
GPe: Globus pallidus external
PM: Pattern memory
E: Empowerment
